# Material discrimination relies on context-dependent active sensing strategies

**DOI:** 10.1167/jov.26.6.4

**Published:** 2026-06-08

**Authors:** Ryu Nomachi, Hideki Tamura, Kevin A. Helgeland, Takuma Morimoto, Shigeki Nakauchi, Tetsuto Minami

**Affiliations:** 1 Department of Computer Science and Engineering, Toyohashi University of Technology, Toyohashi, Japan; 2 Department of Computer Science, Norwegian University of Science and Technology, Trondheim, Norway; 3 Department of Experimental Psychology, University of Oxford, Oxford, UK

**Keywords:** material perception, active sensing, virtual reality, human psychophysics

## Abstract

Material perception is typically studied under passive and highly constrained viewing conditions, thus leaving it unclear whether humans rely on active sampling strategies to resolve perceptual ambiguities. In everyday vision, however, observers naturally move their heads and manipulate objects with their hands to obtain informative cues, thus raising the question of how such exploratory actions contribute to material discrimination. We used immersive virtual reality to examine whether viewpoint changes and object manipulations support the discrimination of visually challenging materials, specifically metal versus glass, whose appearances depend strongly on the associated illumination and viewing geometry. Across three experiments, we found that observers increased the amount of exploratory movements—specifically, viewpoint changes and object manipulations—when the material identity was ambiguous, and that a greater amount of exploration was associated with higher discrimination performance. By independently manipulating head- and hand-based motions, we identified the context-dependent contributions of each movement type; viewpoint changes dominated when multiple objects could be compared simultaneously, whereas object manipulation was more effective when only a single target was available. Moreover, the participants differed in the effectiveness of their sampling strategies, with variation in discrimination sensitivity reflecting both individual differences in strategy and changes over time. Together, these findings demonstrate that material discrimination depends on flexible, context-dependent active exploration rather than passive evaluation of static images. By dissociating head- and hand-based exploration, our study clarifies how observers select actions that generate diagnostic visual information for material discrimination.

## Introduction

Material perception allows humans to identify what objects are made of—such as metal, glass, plastic, or fabric—based on visual information. Although material perception can involve multiple sensory modalities, including haptic perception, the present study focuses specifically on visual material perception. A large body of work has established how mid-level visual processes recover surface properties from the interplay of shading, specular reflections, and three-dimensional shapes (Anderson, 2011; Anderson, 2020; Anderson & Marlow, 2023; Fleming, 2014; Fleming, 2017; Marlow, Kim, & Anderson, 2012). Many studies have investigated these mechanisms using image-computable analyses and computational models based on static images or controlled image statistics (Cheeseman, Ferwerda, Morimoto, & Fleming, 2025; Liao, Sawayama, & Xiao, 2023; Morimoto et al., 2025; Prokott, Tamura, & Fleming, 2021; Prokott & Fleming, 2022; Storrs, Anderson, & Fleming, 2021; Tamura, Prokott, & Fleming, 2022; van Assen, Nishida, & Fleming, 2020). These studies have provided important insights into how the visual system extracts material cues from image structure.

Moreover, other works have demonstrated that many cues supporting material perception are inherently dynamic and changes systematically with illumination conditions (Kim & Marlow, 2016; Xiao et al., 2014), viewpoint (Marlow & Anderson, 2016; Sakano & Ando, 2010; Tani et al., 2013), and object poses (Doerschner et al., 2011; Doerschner, Yilmaz, Kucukoglu, & Fleming, 2013; Kim, Marlow, & Anderson, 2011; Ohara, Kim, & Koida, 2020; Tamura, Higashi, & Nakauchi, 2018; Wendt, Faul, Ekroll, & Mausfeld, 2010). Additionally, studies of specular objects have shown that motion-dependent image transformations, such as specular flow, can strongly influence the perception of three-dimensional shape and surface structure (Dövencioğlu, Wijntjes, Ben-Shahar, & Doerschner, 2015; Dövencioğlu, Ben-Shahar, Barla, & Doerschner, 2017). Such dynamic transformations—including specular flow patterns, highlight motion, and refractive distortions—often provide diagnostic information for distinguishing materials when static images alone are ambiguous.

**Figure 1.**
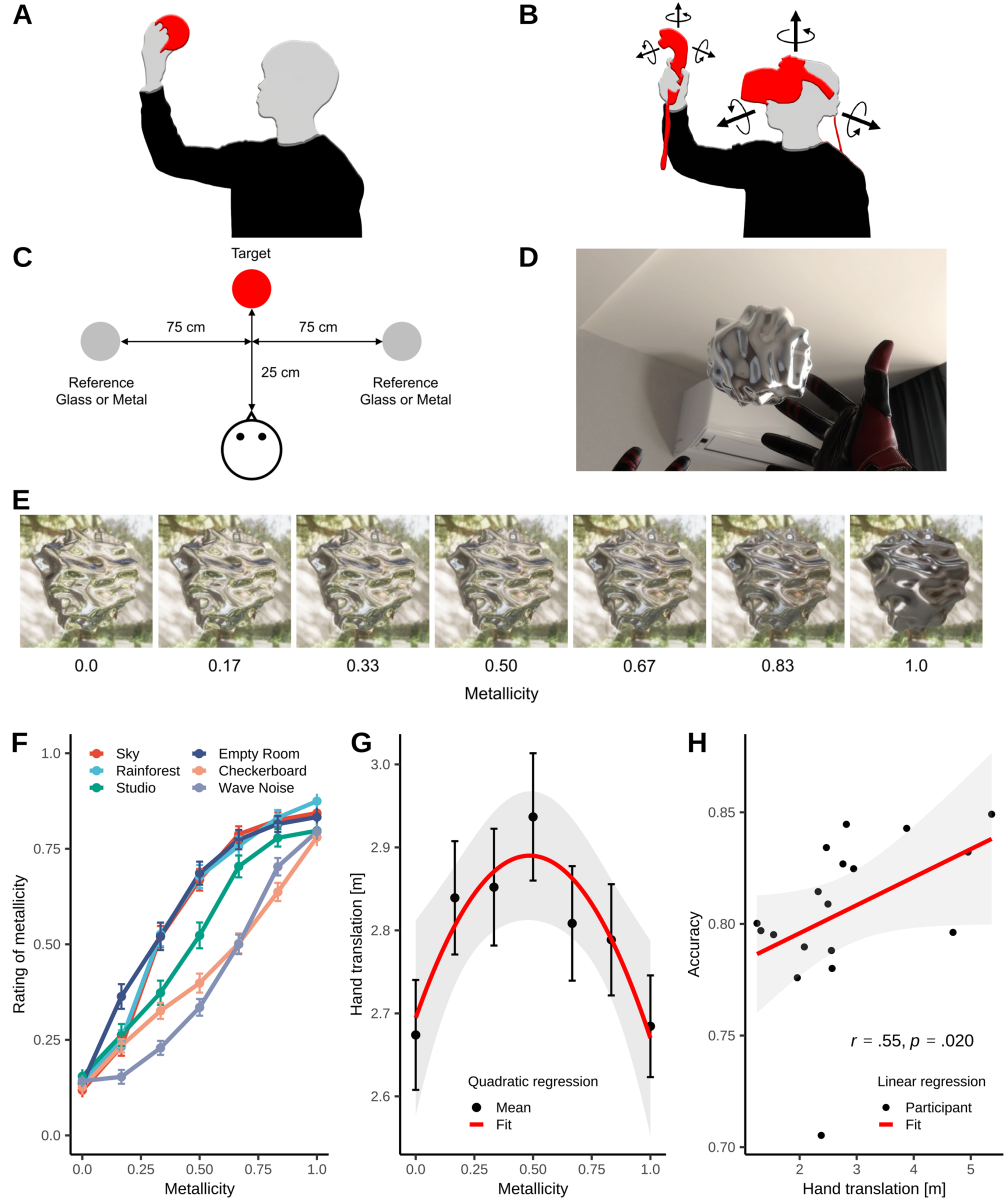
Experimental setup and results of Experiment 1. (**A**) Conceptual illustration of the study, depicting an observer freely exploring objects in a naturalistic environment. (**B**) Virtual-reality implementation of the scenario shown in (**A**). The head and hand positions and orientations of the participants were continuously recorded from a head-mounted display (HMD) and handheld controllers, respectively. (**C**) Schematic diagram of the experimental setup and the spatial arrangement of the stimuli and participants involved in Experiment 1. (**D**) Example of a participant's view in Experiment 1, as rendered within the virtual environment. (**E**) Examples of the experimental stimuli, illustrating a continuum from a glass-like appearance (metallicity = 0.0) to a metal-like appearance (metallicity = 1.0). (**F**) Relationship between physical metallicity and the subjective perceived metallic appearance ratings. Line colors indicate different illumination environments. The error bars denote the standard errors. (**G**) Relationship between physical metallicity and the amount of hand translation. The error bars indicate the standard errors, and the shaded regions represent 95% confidence intervals. (**H**) Relationship between the amount of hand translation and the resulting discrimination accuracy.

Despite this progress, nearly all empirical and theoretical research on material perception has relied on passive or experimenter-controlled viewing conditions. Observers typically remain stationary while objects are rotated for them, or they view brief animations in which motions are predetermined. These paradigms successfully reveal which optical cues can support material recognition, but they leave open a foundational question: How do humans actively sample visual information when they are free to select their own movements? A few recent studies have begun to address aspects of this issue. For example, prior work reported qualitative observations of how observers freely inspect real translucent objects, suggesting that viewing behavior can influence the perceived appearance of materials (Gigilashvili, Thomas, Hardeberg, & Pedersen, 2021). Additionally, other studies have shown that gaze allocation during material perception depends on the perceptual task, indicating that observers actively sample task-diagnostic image regions (Metzger, Ennis, Doerschner, & Toscani, 2024; Toscani, Yücel, & Doerschner, 2019). More broadly, perceptual exploration in natural behavior has been proposed to involve active sampling of task-relevant information through self-generated movements, rather than passive reception of visual input (Goettker, Powell, & Hayhoe, 2025). In natural vision, observers rarely experience objects under fixed or externally controlled conditions. Instead, they continuously move their heads and hands to obtain informative viewpoints (Figure 1A). Such behaviors—approaching an object, rotating it, or peering at it from above or below—fundamentally change the retinal input. However, we know little about how people choose among these candidate actions, how they deploy them under perceptual uncertainty, or how different exploratory modes contribute to material judgments. As a result, we currently lack a principled account of active material perception, although most real-world material judgments depend on self-generated viewpoints and pose changes.

Furthermore, recent work (Lin, Drewing, & Doerschner, 2025) has shown that exploratory movements are tuned to the perceptual task, particularly for glossiness judgments that depend on specular structure information. However, this study does not resolved when and why active exploration becomes beneficial or whether its effectiveness depends on the structure of the viewing environment. It also remains unclear how different exploratory actions—such as viewpoint changes versus object manipulation—contribute to resolving material ambiguities; that is, situations in which different materials produce similar static image structures and therefore require additional dynamic information to be distinguished. Although Lin et al. (2025) demonstrated that exploratory movements are tuned to the perceptual task, the dependence of different exploratory actions on the viewing context and environmental structure remains unclear. Unlike Lin et al. (2025), who focused primarily on hand-based interactions, the present study explicitly dissociates head-driven viewpoint changes and hand-driven object manipulations. More broadly, perception–action coupling theories propose that exploratory movements are not merely outputs of the motor system but rather integral components of perceptual inference (Xiao & Liao, 2025). Whether such principles apply to material perception remains unknown.

Beyond task-dependent exploration, two additional factors may be important for understanding how active sensing supports material perception. In particular, viewpoint changes produced by observer motion and object rotations can provide complementary information for recovering three-dimensional structure (Cornilleau-Pérès & Droulez, 1994; Durgin, Proffitt, Olson, Reinke, 1995; Todd, 2004). Consistent with this idea, more recent studies have shown that viewpoint and motion direction can influence the perception of specular objects and their apparent shape, highlighting the importance of spatial viewing geometry for material appearance (Dövencioğlu et al., 2015; Dövencioğlu et al, 2017). Moreover, previous research has reported substantial individual differences in how observers integrate visual cues when they judge object color and material properties in naturalistic tasks (Brainard, Cottaris, & Radonjić, 2018). Psychophysical studies also suggest that observers may differ in how they interpret visual cues when judging material properties such as translucency (Liao, Sawayama, & Xiao, 2022). These findings raise the possibility that individuals may adopt distinct exploratory strategies when interacting with ambiguous material stimuli.

Therefore the present study addresses this gap by introducing a virtual-reality (VR) framework that allows observers to freely move both their viewpoints and the objects that they are inspecting (Figure 1B). This approach enables fine-grained, frame-by-frame measurements of head and hand movements while preserving ecological validity. Using this framework, we asked three fundamental questions motivated by the dynamic nature of material cues, prior observations of task-dependent exploration, and the possibility that exploratory behavior depends both on viewing geometry and individual strategy differences: (1) Do observers strategically regulate their movements to resolve material ambiguities? (2) How do viewpoint changes (head motions) and object manipulations (hand motions) differentially contribute to the discrimination process? (3) Do individuals exhibit distinct exploratory strategies, and are these strategies associated with differences in material discrimination sensitivity across individuals?

Across three experiments, participants judged whether visually challenging objects were metal or glass because the appearances of these materials are strongly modulated by environmental illumination conditions (Kim & Marlow, 2016; Ohara et al., 2020; Ohara, Kim, & Koida, 2022; Tamura et al., 2018; Tamura et al., 2022; Tamura & Nakauchi, 2018). Metal surfaces produce mirror-like reflections of the surrounding scene (Fleming, Torralba, & Adelson, 2004; Harvey & Smithson, 2021; Muryy, Fleming, & Welchman, 2016; Schmid, Barla, & Doerschner, 2023; Todd & Norman, 2018), whereas glass transmits and refracts light, creating distorted images of the background (Fleming, Jakel, & Maloney, 2011; Gigilashvili, Thomas, Pedersen, & Hardeberg, 2021; Schlüter & Faul, 2014; Schlüter & Faul, 2016; Schlüter & Faul, 2019; Todd & Norman, 2019; Todd & Norman, 2020). Although metal and glass differ in physical properties such as opacity and light transmission, their visual appearances can partially overlap under certain illumination and viewing conditions. In particular, both materials can produce strong specular reflections of the surrounding environment, which may obscure diagnostic refractive cues from glass. Under such conditions, distinguishing between the two materials may be less straightforward than their physical differences might suggest.

Furthermore, by manipulating whether head or hand motions produced visual changes, we dissociated the contributions of distinct modes of exploration. These modes correspond to viewpoint changes produced by head movements and object rotations produced by hand movements, which tend to emphasize different classes of dynamic visual information. This distinction is consistent with prior work showing that viewpoint changes produced by observer motion and object rotations can provide complementary information for recovering three-dimensional structure (Cornilleau-Pérès & Droulez, 1994; Durgin et al., 1995; Todd, 2004). More recent work has further emphasized that visual inference about object motion and depth depends on the viewing geometry generated by self-motion and object motion (Xu, Pang, Anzai, & DeAngelis, 2025). We quantified exploratory behaviors using translation and rotation metrics, and extracted individual strategy profiles using principal component analysis (PCA) and Gaussian mixture model clustering. This allowed us to link how participants moved to how well they perceived objects, revealing the strategic dimensions of active sensing.

Our findings reveal that active explorations are not uniformly beneficial but instead depend critically on the availability of informative dynamic cues. In the present study, these cues likely included changes in specular reflections and refractive distortions generated by viewpoint shifts and object rotations, which are plausible sources of diagnostic information for distinguishing metal from glass. First, we found that active exploration was associated with higher material discrimination performance in a virtual-reality environment, such that larger translation and rotation movements during exploration tended to accompany better identification performance (Experiment 1). Subsequently, viewpoint changes enhanced participants’ discrimination performance when comparative visual information across multiple objects or viewpoints was available (Experiment 2), whereas object manipulation was more effective when such viewpoint cues were limited (Experiment 3). Across experiments, the number of exploratory movements increased selectively under conditions of high perceptual uncertainty, indicating that observers chose actions that reduced ambiguity rather than producing nonspecific motions. Moreover, analyses of the data from Experiments 2 and 3 revealed striking individual differences in exploratory strategies, with more efficient sampling behaviors predicting greater learning-related sensitivity gains across trials. Together, these results show that material discrimination emerges from a flexible, context-dependent perception–action loop rather than from passive evaluations of static images. By combining VR-based behavioral measurements with quantitative analyses of exploratory actions, our study clarifies how observers actively select movements that generate diagnostic visual information for material discrimination.

## General methods

### Participants

Twenty students at the Toyohashi University of Technology participated in each of Experiments 1, 2, and 3. The sample size was determined on the basis of previous virtual-reality material perception studies, which typically included between 12 and 20 participants (Niu & Lo, 2022; Pardo, Suero, & Pérez, 2018). All the participants had normal or corrected-to-normal vision and were naive to the purpose of the experiments. Four individuals participated in both Experiments 1 and 2, whereas there was no overlap between Experiments 2 and 3. The experiments were conducted in a fixed order, with Experiments 1, 2, and 3 performed sequentially. All participants were provided with a full explanation of the experimental procedures and provided written informed consent prior to participating. This study was approved by the Committee for Human Research of the Toyohashi University of Technology.

### Apparatus

The participants were seated in a chair and wore a Varjo Aero head-mounted display (resolution: 2880 × 2720 per eye; field of view: 115°; refresh rate: 90 Hz). They additionally held a pair of VR controllers (HTC Vive controller), one in each hand. Positional tracking was achieved using SteamVR Base Station 2.0. The virtual environments were implemented in Unity (version 2022.3.20f1 for Experiment 1 and 2022.3.22f1 for Experiments 2 and 3) with the Varjo XR Plugin (version 3.7.0) and SteamVR Plugin (version 2.8.0). To perform physically accurate rendering, we used Unity's High-Definition Rendering Pipeline (HDRP). To minimize the degree of simulator sickness, it was essential to maintain a smooth rendering process with minimal latency and flicker. Therefore we adopted a BSDF-based material model. Because the primary visual cues of interest in this study concerned surface reflection and transmission, subsurface scattering was not needed. This modeling choice allowed us to achieve stable, high-fidelity rendering while maintaining high real-time performance.

### Stimuli

A randomly bumped three-dimensional object (8 cm × 8 cm × 8 cm) was presented at a viewing distance of 25 cm in Experiment 1. In Experiments 2 and 3, a geometrically identical but smaller object (6 cm × 6 cm × 6 cm) was presented at a viewing distance of 50 cm. At these initial spatial arrangements, the objects subtended visual angles of 18.19° (Experiment 1) and 6.87° (Experiments 2 and 3), respectively. The materials were implemented using the HDRP Lit shader in Unity, with the following parameters fixed: smoothness = 1.0, index of refraction = 1.424, and thickness = 0.8. The metallic parameter determined the degree to which the surface appeared metallic. Within HDRP's physically based rendering framework, this parameter controls the balance between diffuse transmission and surface reflection by modifying the material's normal-incidence reflectance. This modification shifts the appearance from a dielectric (glass-like) surface dominated by transmission and refraction toward a conductor-like (metallic) surface dominated by surface reflection. By varying this parameter from 0.0 to 1.0, we generated a continuous transition from a glass-like appearance to a metal-like appearance. This parameter served as the metallicity factor. In Experiments 1 and 2, seven levels were used (0.0, 0.17, 0.33, 0.50, 0.67, 0.83, and 1.0), whereas Experiment 3 used five levels (0.0, 0.25, 0.5, 0.75, and 1.0). Thus the stimuli spanned a continuum from transparent glass-like objects to mirror-like metallic objects. Subsurface scattering was disabled to avoid unintended translucent appearances at intermediate levels (see also the “Apparatus” section). Rendering details are provided in the “1. Renderings” section of the Supplemental Information.

To validate the perceptual plausibility of these materials, we conducted a matching experiment in which the participants compared our stimuli to physically based renderings with systematically varied refractive indices (Tamura et al., 2018). The results (*n* = 7) confirmed that stimuli with higher metallicity levels were consistently matched to materials with higher refractive indices (Supplementary Figure S1).

Two object shapes were used on the basis of prior work (Tamura et al., 2018): a relatively smooth object (“smooth”) and a more irregular object (“bumpy”), which was created by randomly modulated surface perturbations (see Supplementary Figure S2A). Surface irregularities were generated procedurally using the mesh subdivision function with fractal modulation, following the procedure described in a previous study (Tamura et al., 2018). These shapes were intentionally designed to be nonsemantic, so that observers relied primarily on visual appearance rather than object identity when making material judgments.

Illumination was provided using 4π-steradian high-dynamic-range-imaging (HDRI) environment maps obtained from Poly Haven (https://polyhaven.com/, CC0 license). The virtual scene consisted of a spherical HDRI illumination dome in which the stimulus object appeared to float directly in front of the observer. Because the HDRI background was rendered as an infinitely distant environment map, observer translations did not change the background perspective. In Experiment 1, two outdoor (Sky and Rainforest) and two indoor (Studio and Empty Room) lighting environments were selected from this database (Supplementary Figure S2B). In addition, two artificial environments generated in Blender were used, including one structured environment (Checkerboard) and one unstructured environment (Wave Noise). Experiments 2 and 3 used two natural HDRI environments (Sky and Studio) and two artificial environments (Check and Gray) (the illumination conditions are shown in Supplementary Figure S2C; example stimuli are presented in Supplementary Figure S2D). These illumination environments were selected to provide diverse environmental structures and reflection patterns, allowing examination of how variations in environmental illumination influence the visual cues available for distinguishing metal from glass. Supplementary Movies S1 and S2 showing the objects within the actual VR scene used in Experiment 2 is provided.

### Data analysis

All analyses were conducted in R (version 4.5.1). In Experiment 1, two participants were excluded because they responded in a binary manner for a substantial portion of the session, indicating that they did not fully understand the continuous slider response system. The final analysis therefore included 18 participants. No participants were excluded from Experiment 2 (*n* = 20). In Experiment 3, one participant was removed because of their inability to obtain proper optical focus while wearing the HMD, resulting in a final sample consisting of 19 participants.

#### Response data

For each trial, participants reported the perceived material of the target stimulus using a continuous slider ranging from “glass-like” to “metal-like,” indicating how close the target appeared to either reference stimulus. Accuracy was computed by normalizing the absolute difference between each participant's rating and the true material value (metallicity) of the stimulus by the maximum possible difference. The accuracy scores were then averaged separately for each participant (Figure 1H).

#### Behavioral data

##### Translational movement

For each condition, we computed the across-participant mean movement distances of the head and right hand. For each trial, the total movement distance *D* was calculated using the following equation:

$$\begin{array}{ccc} D\; & = & \;\sum_{i=2}^{N}\sqrt{{\left(Xp_{i}-Xp_{i-1}\right)}^{2}}+\sum_{i=2}^{N}\sqrt{{\left(Yp_{i}-Yp_{i-1}\right)}^{2}}\\ & & +\sum_{i=2}^{N}\sqrt{{\left(Zp_{i}-Zp_{i-1}\right)}^{2}}, \end{array}$$

where *N* denotes the number of frames within a trial, and *X*p_*i*_,*Y*p_*i*_,*Z*p_*i*_ represent the XYZ coordinates of either the HMD (head) or the experimental stimulus (hand) at frame *i*. The amount of translation was then averaged within each observation condition and used in subsequent analyses.

##### Rotational movement

We computed the mean rotational displacements of the HMD (head) and of the stimulus (hand). For a given trial, the rotational displacement *R* was quantified according to the following equation:

$$\begin{array}{ccc} R\; & = & \;\sum_{i=2}^{N}2\mathrm{co}s^{-1}\left(\right|Wr_{i}Wr_{i-1}+Xr_{i}Xr_{i-1}\\ & & +Yr_{i}Yr_{i-1}+Zr_{i}Zr_{i-1}\left|\right), \end{array}$$

where *N* denotes the number of frames within a trial and $Wr_{i},Xr_{i},Yr_{i},Zr_{i}$ represent the quaternion components of the HMD (or of the stimulus) at frame *i*. The resulting rotational displacement values were averaged within each observation condition for further analysis.

##### Polynomial modeling of exploratory behavior as a function of material ambiguity

To determine whether the participants modulated their exploratory behaviors as a function of material ambiguity, we predicted translational (or rotational) movement using a polynomial regression model as a function of the metallicity of the stimulus. On the basis of the hypothesis that participants would engage in more active information sampling for ambiguous stimuli (e.g., metallicity = 0.50) than for unambiguous stimuli (e.g., metallicity = 0.0 or 1.0), the translational distance *D* was modeled as a function of the linear and quadratic metallicity terms *M*:*D* ∼ *M* + *M^2^*. Similarly, rotational movement *R* was modeled using the same polynomial structure: *R* ∼ *M* + *M*^2^.

## Experiment 1: Active exploration contributes to material discrimination

The goal of Experiment 1 was to determine whether active explorations enhance material discrimination performance when observers freely manipulate an object while judging its material properties. Motivated by previous studies combining virtual reality and material perception (e.g., Lin et al., 2025), the target object appeared directly in front of the observer, with the reference objects, one with metallicity = 0.0 (glass) and the other with metallicity = 1.0 (metal), positioned 90° to the left and right (Figure 1C). By pressing the trigger on the controller in one hand, the participants could virtually “grasp” the target and move it freely in space. Under two object shapes (Supplementary Figure S2A) and six illumination environments (Supplementary Figure S2B), participants judged how similar the target appeared to each reference material using a continuous slider (the participants’ point of view is shown in Figure 1D). Metal and glass were chosen because their appearances depend strongly on illumination and viewing geometry conditions. Metals primarily convey their identity through specular reflections of the surrounding environment, whereas glass reveals refractive and transmitted light patterns. These properties make the metal–glass distinction a demanding perceptual judgment task, providing a suitable test case for examining how active explorations support material discriminations (example stimuli are presented in Figure 1E).

### Methods

#### Procedure

Each participant sat on a chair while wearing the HMD and holding a controller in each hand. A target object was presented 25 cm in front of the participant. Two reference objects—one fully glass object (metallicity = 0.0) and one fully metal object (metallicity = 1.0)—were placed 75 cm to the left and right of the target (Figure 1C), respectively. This arrangement prevented the target and the reference objects from being visible simultaneously. The participants used the trigger on the right-hand controller to virtually grasp the target object and freely translate and rotate it. Hand movements were rendered in real time using a glove-shaped virtual avatar linked to the controller. Both virtual hands were visible and moved in accordance with the participant's controller input, but only the right hand could grasp and manipulate the target object. When the object was grasped, the hand model was no longer rendered and only the object remained visible during manipulation, preventing potential occlusion of the stimulus by the virtual hand.

To provide haptic confirmation during manipulations, the controller delivered a brief vibration whenever the virtual hand made contact with the object. This vibration occurred once at the moment of contact and did not continue while the hand remained in contact with the object. The grasp state was triggered when the participant pulled the trigger while the virtual hand was within a predefined distance of the object, allowing the object to be translated and rotated using the controller.

Participants were instructed to freely explore the target object by translating and rotating it after it appeared in the scene, and to judge whether its material appearance was closer to the glass or metal reference. The judgment was reported using a slider bar controlled by the left-hand controller. Participants were required to observe the stimulus for at least six seconds before they responded. Responses were disabled during this initial period to prevent immediate judgments based solely on a single static view. This duration was determined on the basis of pilot testing to allow sufficient time for exploratory interactions. After this initial period, participants could continue exploring the object for as long as they wished before making their response. Additionally, participants performed the task while seated and were not allowed to walk around the scene. Thus they could not physically move behind the object to inspect it from the rear. The object could be translated and rotated within the reachable workspace but could not pass through other objects in the scene. Each participant completed 168 trials (7 metallicity levels × 2 shapes × 6 illumination environments × 2 reference-position configurations: glass-left/metal-right vs. metal-left/glass-right). The participants were allowed to take a break following every 42 trials. Five practice trials were conducted before the main experiment.

#### Data analysis

For each participant and condition, the corresponding metallicity rating was analyzed using a linear mixed-effects model implemented in the lme4 package (version 1.1.37). The model included the metallicity rating *R* as the dependent variable, with metallicity *M* and illumination *E* as fixed effects and as an interaction term, and the participant ID and shape *G* as random effects. Shape was modeled as a random effect because it was introduced to increase stimulus variability rather than as a factor of theoretical interest. The model was specified as: *R* ∼ *M* × *E* + (1 + *M*|*ID*) + (1|*G*). Effect sizes are reported as likelihood-based estimates of partial eta squared ($\eta_{p}^{2}$) values for the linear mixed-effects model (Figure 1F), which were computed using the eta_squared function from the effectsize package.

### Results

An analysis of the rating data (Figure 1F) revealed that higher physical metal contents led to higher perceived metallicity levels (*F*(1,  17) = 139.56, *p* < 0.001, $\eta_{p}^{2}=0.891$), and that the judgments differed across illumination environments (*F*(5,  2977) = 18.13, *p* < 0.001, $\eta_{p}^{2}=0.03$). We also observed a significant interaction between the material and illumination (*F*(5,  2977) = 4.25, *p* < 0.001, $\eta_{p}^{2}=0.007$), indicating that the degree to which an object appeared metallic—or glass-like—depended strongly on the surrounding light field. Specifically, objects tended to be perceived as more metallic under natural illumination, whereas the same objects were more likely to be perceived as glass-like under artificial illumination. These results confirmed that, even under active viewing, observers could reliably discriminate between materials in a manner consistent with that observed in previous passive viewing studies.

Turning to the participants’ exploratory behaviors, we found that the amount of object translation (see Data analysis) exhibited a clear inverted U-shaped relationship with the physical material along the metal–glass continuum (Figure 1G): the degree of translation was greatest at metallicity = 0.50, where the stimulus was most ambiguous (*β* = −0.83, *p* = 0.002). This relationship was also observed within each illumination condition individually. These results suggest that observers increased their exploratory movements when perceptual uncertainty about the material was higher.

Similarly, greater object translation was associated with higher overall discrimination accuracy, defined as the normalized agreement between each participant's rating and the true metallicity of the stimulus, yielding a positive correlation between exploration and performance (Figure 1H; *r* = 0.55, *p* = 0.020). Together, these findings suggest that active viewing allows observers to adopt different sampling strategies and reveal an association between exploratory extent and material discrimination performance.

## Experiment 2: Dissociating the contributions of viewpoint motions and object manipulations

The exploratory behavior observed in Experiment 1 combined two sources of movement: viewpoint changes produced by head motions and object manipulations produced by hand motions. Therefore it was unclear which mode-specific cues were primarily responsible for the material discrimination performance observed in the results. Moreover, the correlation between exploratory movements and discrimination performance observed in Experiment 1 does not establish a causal relationship. Experiment 2 was designed to examine how restricting different forms of exploration affects material discrimination performance by dissociating the respective contributions of head- and hand-based motion signals. Previous work has demonstrated that active explorations can benefit material judgments and that observers generally conduct more extensive explorations during gloss judgments than during lightness judgments (Lin et al., 2025). However, the specific movements that drive this benefit—and how their usefulness depends on the illumination environment—remain unresolved.

**Figure 2.**
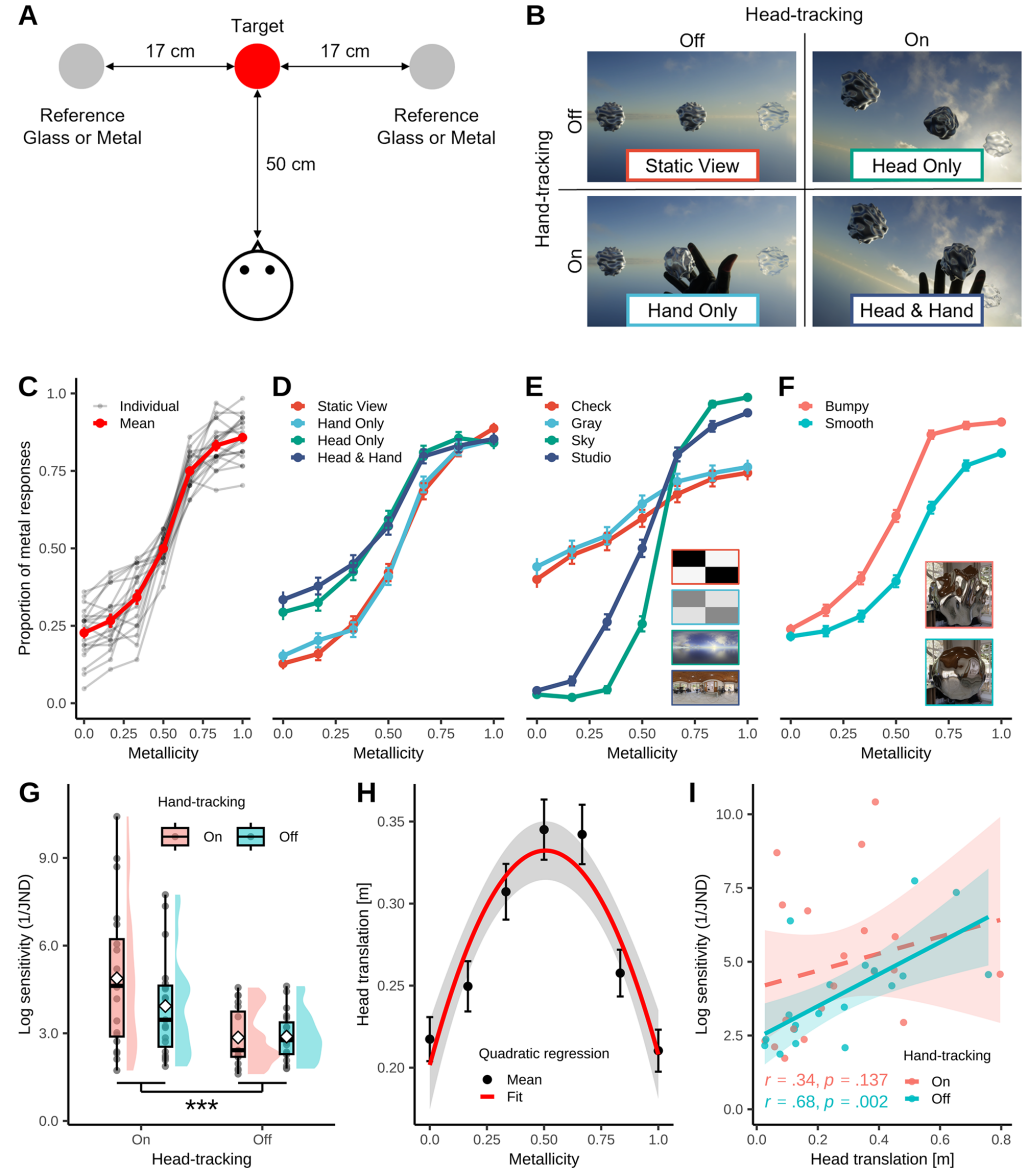
Results of Experiment 2. (**A**) Schematic illustration of the experimental setup and the spatial arrangement of the stimuli and participants involved in Experiment 2. (**B**) Experimental conditions and example views from the participants’ perspective in the virtual environment. (**C**) Relationship between physical metallicity and the subjective metal responses. The red line indicates the group mean, and the light gray lines represent individual participants’ data. (**D–F**) Same data as those shown in (**C**), replotted separately by experimental condition, illumination, and object shape. (**G**) Discrimination sensitivity (log-transformed 1/JND) across different experimental conditions. White diamonds denote group means, and gray points indicate individual participants. (**H**) Relationship between physical metallicity and the amount of head translation. The formatting is identical to that utilized in Figure 1G. (**I**) Relationship between the amount of head translation and the resulting discrimination sensitivity. Asterisks indicate significant differences: ***p < 0.001.

To address this gap, Experiment 2 dissociated the contributions of head- and hand-based exploration by selectively enabling or disabling head and controller tracking. Using a setup similar to that used in Experiment 1 but with the reference objects positioned on both sides of the target (Figure 2A), we tested all combinations of head motion tracking (on/off) and controller tracking (on/off), yielding four conditions that isolated the effects of viewpoint motions and object manipulations (Figure 2B). The participants performed a 2AFC task to determine whether the target appeared more similar to the left or right reference material (metal or glass) while varying the viewpoint, the object, both, or neither. The illumination conditions included two natural light fields and two artificial fields to examine whether the usefulness of each mode of exploration depended on the environmental context.

### Methods

#### Procedure

The target stimulus was presented 50 cm in front of the participant and was flanked by two reference stimuli positioned 17 cm to the left and right (Figure 2A). At trial onset, all three stimuli were visible simultaneously from a single central viewpoint, allowing participants to directly compare the target with the two references. Participants indicated which reference the target most closely resembled using a button located on the left-hand controller. Participants were allowed to explore the object freely, and no explicit time limit was imposed on the exploration period. The degree of observational freedom was manipulated by orthogonally combining the presence or absence of head motions (HMD tracking enabled or disabled, respectively) and hand motions (controller tracking enabled or disabled, respectively), yielding four experimental conditions (Figure 2B). Under the hand-motion-available conditions, the participants could grasp and freely manipulate the target stimulus with their right hands. The reference stimuli were either fully glass-like (metallicity = 0.0) or fully metal-like (metallicity = 1.0), and the left–right assignment of these reference stimuli was counterbalanced and treated as the reference position factor.

Each participant completed 224 trials per day (4 observational conditions × 7 metallicity levels × 2 shapes × 2 reference-position configurations × 2 illumination conditions). The trials were blocked by observational condition, with each block consisting of 56 trials (7 metallicity levels × 2 shapes × 2 reference-position configurations × 2 illumination conditions), resulting in four blocks per day. The order of the blocks was counterbalanced across participants, and the order of trials within each block was randomized. The experiment was conducted over two separate days, yielding a total of 448 trials per participant. The four illumination conditions (Supplementary Figure S2C) were divided across the two days, such that the two natural illumination conditions were tested on one day and the two artificial illumination conditions on the other. The order of these sessions was counterbalanced across participants. Before each session, participants completed a short practice session consisting of eight trials (4 observational conditions × 2 illumination conditions).

#### Data analysis

Psychometric functions were fitted to each participant's response data as a function of stimulus metallicity for each observational condition using a probit model. The point of subjective equality and the slope parameter (*σ*) were derived from the fitted functions. The just-noticeable difference (JND) was defined as half the difference between the 75% and 25% response points of the fitted psychometric function, corresponding to an interquartile-based estimate of discrimination threshold. Trials in which the estimated JND exceeded the interquartile range were excluded from subsequent analyses (exclusion rate: 3.75%).

To quantify the degree of material discriminability, discrimination sensitivity was defined as the inverse of the JND (1/JND). Because sensitivity values are typically positively skewed and reflect multiplicative changes, they were log-transformed before statistical analysis. This transformation is commonly used in psychophysical research to stabilize variance and improve the normality of model residuals.

We then fitted a linear mixed-effects model to predict log-transformed sensitivity *S*, with *Head* (presence vs. absence of head movement) and *Hand* (presence vs. absence of hand movement) as fixed effects and their interaction and participant identity *ID* as a random intercept. The model was specified as: *S* ∼ *Head* × *Hand* + (1 | ID). Fixed effects were tested using Type III ANOVA with Satterthwaite's approximation for the degrees of freedom. Effect sizes are reported as approximate partial eta squared values derived from the *F* statistics.

### Results

An analysis of the choice data (Figure 2C) revealed that a higher physical metal content again led to higher metal-response rates, replicating the results of Experiment 1. When the results were broken down by viewing condition, the participants were more likely to judge the stimuli as metallic when head motions were available (Figure 2D). The illumination level also modulated the responses: artificial light fields yielded more ambiguous judgments than natural fields did (Figure 2E). In addition, objects with deeper surface reliefs were more often judged as metallic (Figure 2F). Together, these results indicated that the material judgments depended strongly on the observer movements, illumination conditions, and shapes.

We subsequently examined the discrimination sensitivity across the four viewing conditions (Figure 2G). A significant main effect of head motion was observed ($F\left(1,\;54.66\right)=24.81,\;p<0.001,\eta_{p}^{2}=0.312$), whereas the main effect of hand motion was not significant ($F\left(1,\;54.66\right)=1.15,\;p=0.287,\eta_{p}^{2}=0.021$), and no interaction was detected ($F\left(1,\;54.66\right)=1.88,\;p=0.176,\eta_{p}^{2}=0.033$). Thus, in this context—where participants could observe the target and reference stimuli simultaneously—the cues obtained through viewpoint changes played a dominant role. Similar to Experiment 1, the numbers of both head and hand translations peaked when the physical material was most ambiguous, indicating increased exploratory behavior under uncertainty (*β* = −0.51, *p* < 0.001 for head translation; Figure 2H). Crucially, more movements did not uniformly improve the resulting performance. A strong positive correlation was found between the head translation distance and discrimination sensitivity only when hand motions were disabled (*r* = 0.68, *p* = 0.001; Figure 2I). When hand motions were available, this correlation weakened (*r* = 0.34, *p* = 0.137; Figure 2I). A similar pattern emerged for head rotations (no-hand condition: *r* = 0.70, *p* < 0.001; hand condition: *r* = 0.26, *p* = 0.274).

These results indicate that, in Experiment 2, viewpoint motion served as the primary exploratory strategy because it provided simultaneous access to cues from both the target and reference objects. Importantly, although all objects were visible at the same time, small viewpoint changes altered the viewing geometry and thereby modified the spatial configuration of reflections and refractions on the object surfaces. For reflective or transparent materials, such viewpoint-dependent changes in specular highlights or background distortions can provide additional diagnostic information for material discrimination. Although the present analyses do not identify the specific visual cues that observers relied on, the results suggest that viewpoint motion enabled participants to sample visual information that systematically varies with viewing angle.

## Experiment 3: When viewpoint changes provide limited information, observers rely on object manipulation instead

**Figure 3.**
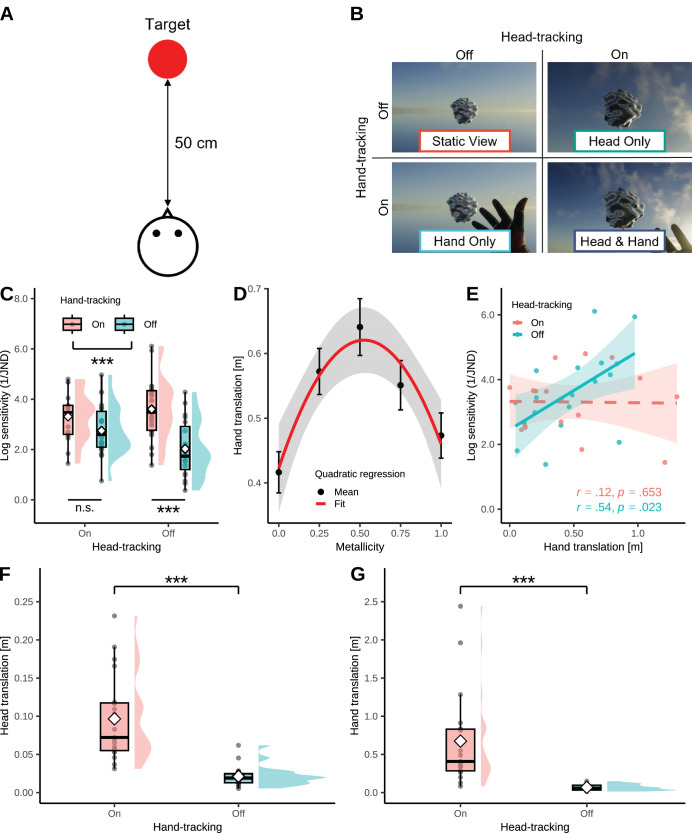
Results of Experiment 3. (**A**) Schematic illustration of the experimental setup in Experiment 3. (**B**) Experimental conditions and example participant-view images in Experiment 3. (**C**) Relationships between the experimental conditions and the discrimination sensitivity (log-transformed 1/JND). (**D**) Relationship between the degree of metallicity and the amount of hand translation. (**E**) Relationship between the amount of hand translation and the resulting discrimination sensitivity. Panels (**C–E**) follow the same format as used in Figures 2G–I. (**F**) Head translation in the head-tracking-off condition (i.e., “empty” head movements). (**G**) Hand translation in the hand-tracking-off condition (i.e., “empty” hand movements). Notably, the identical test statistics in (**F**) and (**G**) resulted from the fact that the Wilcoxon signed-rank tests were applied to two measures derived from the same paired observations, resulting in the same rank ordering across participants. Asterisks indicate significant differences: *p < 0.05, **p < 0.01, ***p < 0.001.

What strategies do observers adopt when viewpoint changes fail to provide sufficient diagnostic information? To address this question, in Experiment 3, the reference objects used in Experiment 2 were removed, leaving only the target object in view (Figure 3A). The participants performed a binary classification task to judge whether the target appeared metallic or glass-like while head-motion tracking and hand-motion tracking were independently enabled or disabled, as in Experiment 2 (Figure 3B). In this configuration, viewpoint motions could no longer yield comparative cues from the reference objects, reducing the relative utility of head-based explorations. If the participants continued to rely on viewpoint changes despite this limitation, it would imply that head motion constitutes a fundamentally important exploratory mode for active material discrimination tasks. Conversely, if increased object manipulation was accompanied by higher discrimination sensitivity in conditions where hand movements were available, this would indicate that observers flexibly shift their exploratory strategy depending on which cues are most informative in the given context.

### Methods

#### Procedure

The procedure was largely identical to that of Experiment 2, except that participants judged whether the target appeared more like metal or glass (Figure 3A). As in Experiment 2, participants were allowed to explore the object freely, and no explicit time limit was imposed on the exploration period. Each participant completed 160 trials (4 observational conditions × 5 metallicity levels × 2 shapes × 4 illumination conditions). The trials were blocked by observational condition, with each block consisting of 40 trials (5 metallicity levels × 2 shapes × 4 illumination conditions), resulting in four blocks in total. The order of the blocks was counterbalanced across participants, and the order of trials within each block was randomized.

Before the main experiment, participants completed a practice session consisting of 16 trials (4 observational conditions × 4 illumination conditions). During the practice session, half of the stimuli were rendered as fully glass objects (metallicity = 0.0) and the other half were rendered as fully metal objects (metallicity = 1.0), providing perceptual anchors for the subsequent categorization task. Glass and metal stimuli were randomly assigned so that each category appeared equally often across trials.

We additionally recorded head and hand movements even when head tracking or hand tracking was disabled. Under these “tracking-off” conditions, movements made by the HMD or controller did not affect the visual scene, meaning that the stimulus did not respond to the participants’ actions. Nevertheless, the participants often moved their heads or hands, and these “empty” exploratory movements were fully logged for analysis purposes (see also Figures 3F and 3G). Participants were not informed that their head and hand movements were being recorded or analyzed.

#### Data analysis

Data analysis was performed in the same manner as that used in Experiment 2. The exclusion rate based on the JND criterion was 5.26%. Post hoc pairwise comparisons were conducted using estimated marginal means (emmeans), with *p*-values adjusted using the Bonferroni correction.

### Results

We observed similar patterns in that experimental conditions, illumination values, and object shape influenced metal responses even when only the target object was presented (see Supplementary Figures S2C and S2D for the illuminations and stimuli, respectively; see Supplementary Figures S2E–H for the results). An analysis of discrimination sensitivity (Figure 3C) revealed a pattern distinct from that observed in Experiment 2. Specifically, whereas head motion showed a significant main effect in Experiment 2, no significant main effect of head motion was found in Experiment 3 ($F\left(1,\;48.29\right)=2.62,p=0.112,\eta_{p}^{2}=0.051$). In contrast, hand motion exhibited a significant main effect ($F\left(1,\;49.92\right)=17.67,p<0.001,\eta_{p}^{2}=0.261$), and a significant interaction between head and hand motion was also observed ($F\left(1,\;48.38\right)=4.98,p=0.030,\eta_{p}^{2}=0.093$). Multiple comparisons revealed that hand motion increased the discrimination sensitivity when head motion was disabled (*t*(50.92) = 4.62; 95% confidence interval [CI], 0.28–1.09; *p* < 0.001; *d* = 0.64), whereas no such benefit was observed when head motion was enabled (*t*(52.22) = 1.38; 95% CI, −0.21 to 0.63; *p* = 0.999; *d* = 0.19). Thus, in contrast with Experiment 2, hand-based object manipulation affected to the material discriminability in Experiment 3 only under conditions in which viewpoint-based cues were weakened. In support of this interpretation, the object translation distance increased when the physical material was most ambiguous, indicating greater reliance on manipulation-based explorations (β = −0.72, *p* < 0.001; Figure 3D). Moreover, greater object movement was associated with higher discrimination sensitivity, but only when hand motion was available and head motion was disabled (*r* = 0.54, *p* = 0.021; Figure 3E). This relationship was not observed when both head and hand motions were enabled (*r* = 0.12, *p* = 0.653; Figure 3E). These findings suggest that observers preferentially rely on object manipulation when it provides sufficiently diagnostic information, demonstrating flexible, context-dependent sampling strategies.

Curiously, when tracking for one exploratory mode was enabled, the number of movements in the other mode increased even when its tracking process was disabled (head translation: *V* = 190, *Z* = 4.62, *p* < 0.001, *r* = 0.75 in Figure 3F; hand translation: *V* = 190, *Z* = 4.62, *p* < 0.001, *r* = 0.75 in Figure 3G). In other words, under conditions where informative cues were limited, participants produced “empty” head or hand motions—movements that did not affect the visual stimulus—suggesting an effort to obtain missing information even when such actions were not visually effective.

## Individual differences in exploratory strategies predict discrimination performance

The results thus far have indicated that the observers flexibly switched between head-based and hand-based sampling strategies depending on the viewing context. To examine how such exploratory strategies differed across individuals when both forms of interaction were available, we focused the following analysis on the head + hand exploration condition. We next examined the extent to which these exploratory behaviors varied across individuals and which specific movement patterns were most conducive to material discrimination. To quantify these differences, we categorized each participant's viewing behavior in terms of four components: “pulling the object closer,” “approaching the object,” “viewing the object from below,” and “viewing the object from above” (see Supplementary Figure S3A for Experiment 2 and Supplementary Figure S3B for Experiment 3). By reference to these behavioral metrics, we classified the participants according to the exploratory strategies they used during the task.

### Methods

#### Classification of exploratory behaviors

The participants’ exploratory behaviors were categorized into four types, and the time spent in each category was quantified (Supplementary Figures S3A and S3B). Because head movements were tracked with six degrees of freedom, participants could change their viewpoint not only by rotating but also by translating their head in three-dimensional space, allowing them to observe the object from slightly above or below even when object manipulation was disabled. Behavioral labels were assigned on a frame-by-frame basis according to whether each frame satisfied the predefined criteria for the corresponding exploration category. “Pulling the stimulus” was defined as instances in which the stimulus–head distance was 40 cm or less and the stimulus had moved farther from its initial position than the participant's head, indicating that the object was actively drawn toward the observer. “Approaching the stimulus” was identified when the stimulus–head distance was likewise within 40 cm, but the participant's head had moved farther from its initial position than the stimulus, which was consistent with self-motion toward the object rather than object manipulation. “Viewing from below” was assigned when the stimulus was positioned more than 5 cm above eye level, indicating observations from underneath. Conversely, “viewing from above” was defined as occurring when the participant's head was more than 5 cm higher than the stimulus, reflecting observations from an elevated viewpoint.

#### Dimensionality reduction and clustering of exploratory strategies

To identify the principal dimensions underlying individual differences in exploratory behavior differences, we performed PCA on the time spent in each of the four exploratory categories defined above separately for Experiments 2 and 3. The first two principal components were retained for further analysis. Gaussian mixture model (GMM) clustering was then applied to the PCA scores to classify the participants into distinct groups on the basis of their exploratory strategies. Cluster membership derived from this analysis was subsequently treated as a categorical factor representing participant groups with distinct exploratory strategies. The optimal number of clusters was determined to be three for both Experiments 2 and 3.

**Figure 4.**
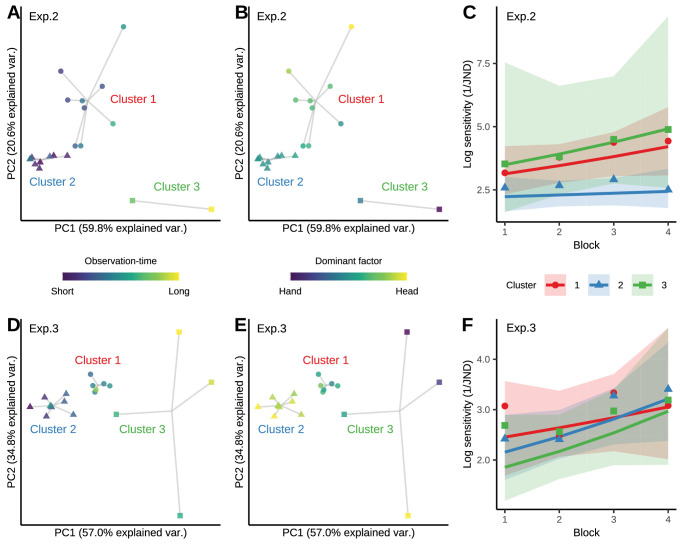
Individual differences among the exploratory strategies used in Experiments 2 and 3. (**A–B**) Results of PCA applied to participants’ exploratory behaviors in Experiment 2. Each point represents one participant, and the marker shapes denote cluster membership. Points are color-coded according to their (**A**) overall observation times and (**B**) relative reliance on head movements versus hand movements. (**C**) Discrimination sensitivity changes observed across different experimental blocks for each PCA-defined cluster in Experiment 2. The experiment was divided into four successive blocks from beginning to end. Shaded regions indicate 95% confidence intervals around the regression lines. (**D–F**) PCA results for the exploratory behaviors observed in Experiment 3. The format is identical to that used in (**A–C**).

The exploratory strategies were visualized on the basis of the scores of the first and second principal components (Figures 4A and 4B for Experiment 2; Figures 4D and 4E for Experiment 3). The data points were color-coded according to (1) the overall observation time and (2) the relative reliance on head-based versus hand-based exploration, which were quantified from both sets of raw observation data (Supplementary Figures S3A and S3B).

To examine changes in performance across clusters, discrimination sensitivity was computed for each participant in each of four successive trial blocks obtained by dividing the full experiment into quartiles, using the head + hand condition. Changes in sensitivity across blocks were then analyzed using a linear mixed-effects model. Log-transformed sensitivity was used as the dependent variable, with blocks and clusters as fixed effects and participant as a random intercept. The model was defined as follows: *S* ∼ *B* × *C* + (1|*ID*), where *S* denotes log-transformed discrimination sensitivity, *B* denotes the block, and *C* denotes the cluster.

### Results

An analysis of Experiment 2 conducted under conditions in which head-based exploration was most informative (Figure 2G) revealed marked individual differences in exploratory strategies. A PCA followed by GMM clustering (Figures 4A and 4B) indicated that participants varied primarily along two dimensions: their overall observation times (PC1; 59.8% of the explained variance) and the relative balance between head- and hand-based explorations (PC2; 20.6% of the explained variance). On the basis of these dimensions, the participants were classified into three distinct clusters. An increase in discrimination sensitivity across four successive trial blocks for each cluster is shown in Figure 4C. A two-way mixed-effects analysis with block and cluster factors revealed a significant main effect of cluster (*F*(2,  16.23) = 5.43, *p* = 0.016, $\eta_{p}^{2}=0.401$) but no main effect of block (*F*(1,  54.05) = 1.35, *p* =  0.251, $\eta_{p}^{2}=\;0.024$) or no block × cluster interaction (*F*(2,  53.42) = 0.25, *p* = 0.783, $\eta_{p}^{2}=0.009$). These results indicate stable sensitivity differences among clusters rather than systematic learning across blocks. To further illustrate these strategy differences, representative head movement trajectories for selected participants are provided in Supplementary Figure S4, demonstrating qualitatively distinct exploration patterns across individuals (see Supplemental Information 5 (Example trajectories of head and hand movements”).

Visual inspection of the data (Figure 4C) revealed distinct patterns across clusters. Cluster 1 showed a gradual increase in sensitivity across blocks, accompanied by relatively narrow confidence intervals, indicating consistent performance across participants, although this trend was not statistically significant. In contrast, Cluster 2 exhibited overall lower discrimination sensitivity across all blocks. The participants in this cluster showed relatively short exploration times and no clear preference for specific exploratory actions, suggesting low strategic consistency. Cluster 3, on average, showed relatively high discrimination sensitivity; however, this cluster was characterized by substantially wider confidence intervals, reflecting greater variability across participants. Notably, this cluster included only two participants; thus, these results should be interpreted with caution. Together, these findings suggest that both the level and stability of sensitivity vary across clusters, highlighting individual differences in exploratory strategies and their relationship to material discrimination.

Similarly, in Experiment 3, PCA followed by GMM clustering revealed a two-dimensional structure underlying individual differences in exploratory behavior, indicating that comparable factors accounted for variability across participants (Figures 4D and 4E). In contrast to Experiment 2, discrimination sensitivity increased significantly across blocks (*F*(1,  66.00) = 4.13, *p* = 0.046, $\eta_{p}^{2}=0.059$; Figure 4F), whereas no significant main effect of cluster was observed (*F*(2,  66.00) = 0.42, *p* = 0.660, $\eta_{p}^{2}=0.013$) and no block × cluster interaction was found (*F*(2,  66.00) = 0.16, *p* = 0.854, $\eta_{p}^{2}=0.005$). This pattern suggests that sensitivity increased over time across participants, regardless of their initial exploratory strategy. These results are consistent with the possibility that observers adjusted their behavior over the course of the experiment, although the present data do not allow us to directly determine the underlying mechanisms of this change.

## Discussion

A large body of work has established the foundations of material perception, describing how mid-level visual processes recover surface properties from the interplay of shading, specular reflections, and three-dimensional shapes (Anderson, 2011; Anderson, 2020; Anderson & Marlow, 2023; Fleming, 2014; Fleming, 2017; Marlow et al., 2012). These frameworks have largely focused on image-based cues, emphasizing how surface properties can be inferred from static patterns of shading, specular reflections, and three-dimensional shape. Nevertheless, many material cues also vary systematically with changes in viewpoint, object pose, and shape (e.g., perception of gloss: Doerschner et al. (2011); viscosity: van Assen, Barla, and Fleming (2018); elasticity: Paulun, Schmidt, van Assen, and Fleming (2017)). Many of these dynamic cues, however, have been characterized using stimuli in which motion is generated by the experimenter rather than the observer. Such viewpoint-dependent transformations—including changes in specular flow, highlight motion, and shading changes—provide powerful information for discriminating among material categories. However, most of this work has relied on passive or experimenter-controlled optical structure changes, raising the question of how observers actively select actions to acquire diagnostic cues.

### Active viewing fundamentally shapes the material discrimination

Our three experiments demonstrate that active viewing fundamentally shapes the human material discrimination process. Allowing observers to freely move their heads and hands increased the flexibility with which they sampled diagnostic visual information, and their discrimination performance improved when these exploratory actions yielded informative cues (Figures 1G and 1H). By dissociating the contributions of viewpoint changes and object manipulations, we found that head motion enhanced the discrimination results when comparative cues were available (Figure 2G), whereas hand-based object manipulations played a dominant role when viewpoint cues were limited (Figure 3C). In both cases, greater use of the exploratory mode that provided useful information was associated with higher discrimination accuracy (Figures 2I and 3E). Clustering analyses further revealed that participants who engaged in more consistent sampling strategies achieved greater discrimination sensitivity overall (Figure 4C) and demonstrated notable flexibility in terms of maintaining their performance even when they relied on distinct exploratory strategies (Figure 4E). In this context, the observed dissociation between head- and hand-based exploration may reflect differences in the primary sources of dynamic visual changes associated with each mode of exploration. Together, these findings reveal that material discrimination is supported by context-dependent exploratory strategies—an aspect of visual perception that is largely undetectable under conventional passive-viewing paradigms.

A recent study has examined whether active exploration can enhance material perception. In particular, Lin et al. (2025) explicitly tested this idea and reported that observers’ exploratory movements were tuned to their perceptual judgments when they were allowed to freely manipulate objects. Exploratory movements were particularly extensive—and presumably more informative—during gloss judgments relative to lightness judgments (Lin et al., 2025). However, their findings did not specify when active exploration becomes beneficial, which exploratory modes drive this improvement, or how these contributions depend on the viewing context. In particular, whereas Lin et al. demonstrated that exploratory behavior varies as a function of the perceptual task while the stimulus remains constant, the present study instead examines how exploratory strategies vary as a function of stimulus properties while the task remains fixed. By systematically separating head- and hand-based cues and comparing conditions in which viewpoint or manipulation information was more or less useful, our experiments demonstrated that the effectiveness of active exploration is highly context-dependent. Crucially, the number of exploratory movements increased specifically under conditions of heightened sensory ambiguity, indicating that observers selected actions to reduce their perceptual uncertainty. This interpretation suggests that the visual system flexibly adapts its sampling strategy to obtain the most diagnostic information available—an idea consistent with theories concerning adaptive, goal-directed perception (Xiao & Liao, 2025). Notably, when one mode of exploration was disabled, the participants often produced “empty” head or hand movements that did not influence the stimulus (Figures 3F and 3G), revealing a strategic intent to seek missing cues even when such movements had no visual consequences. This pattern is in line with the perception–action loop framework, which holds that exploratory behaviors constitute an integral part of perceptual inference rather than ancillary motor outputs.

To rule out alternative explanations for these results, we conducted additional control analyses. First, we examined whether the order of blocks influenced discrimination performance. Linear mixed-effects analyses including block order as a factor showed that, in Experiment 2, the main effect of Head remained significant, indicating that the original conclusion was unchanged. In Experiment 3, we observed significant main effects of Head, Hand, and Block, as well as a significant Head × Hand interaction. Thus, although overall sensitivity varied across blocks, the critical interaction between Head and Hand remained unchanged. These results suggest that the observed effects cannot be attributed solely to learning or order-related biases across blocks.

We also considered the possibility that participants might adopt a simple strategy of moving the object to check whether the background became visible through it. However, analyses of hand–object configurations indicated that such behaviors occurred only rarely (approximately 2% of trials in Experiment 3), suggesting that participants seldom relied on this strategy (see the Supplemental Information 6 “Detection of ‘behind-the-object’ exploration strategy”). Together, these analyses indicate that the observed relationship between exploratory behavior and discrimination performance cannot be readily explained by block-order effects or by the use of such simple heuristic strategies.

### Context-dependent contribution of head- and hand-based explorations

How do observers determine which exploratory action to use when identifying a material? Across our experiments, head motions and hand motions contributed to the discrimination process in different viewing contexts, suggesting that each action can emphasize different diagnostic cues. The viewpoint changes produced by head movements yield specular-flow and motion-parallax information, which becomes particularly informative when multiple objects can be compared simultaneously (Marlow & Anderson, 2016; Sakano & Ando, 2010; Tani et al., 2013). Importantly, manual object manipulation not only generates specular-flow and motion-parallax cues by altering the spatial and temporal distribution of specular highlights but also changes the pose of the object, revealing different highlight configurations (Doerschner et al., 2011; Doerschner et al., 2013; Kim et al., 2011; Wendt et al., 2010). Such exploration-induced changes in highlight structure may serve as diagnostic cues for judging metallicity. In addition, object manipulation may alter refractive and reflective configurations (Ohara et al., 2020; Tamura et al., 2018). In particular, previous work has shown that the perception of mirror versus glass can depend on how optic flow patterns arise as a function of object rotation (Tamura et al., 2018). In the present study, we observed similar patterns when we used not only translational but also rotational measures of exploratory behavior, suggesting that these dynamic cues may have contributed to material discrimination (see also Additional analyses below). This interpretation is consistent with the idea that active exploration functions to generate diagnostic visual information rather than merely to sample it.

Even when both objects are visible from a single viewpoint, head movements dynamically alter the relative geometry between the observer, the object, and the illumination, thereby modulating image-based cues such as specular highlight motion and depth-dependent distortions. Consistent with this account, observers exhibited a bias toward viewpoints from below (Supplementary Figures S3A and S3B), suggesting that they actively sought viewpoints that enhance the visibility of such diagnostic cues. Our additional analyses (Supplementary Figures S5 and S6) further illustrate that object manipulation can give rise to structured temporal changes in image and motion features, including specular highlights and optic flow (Doerschner et al., 2011). In particular, temporal changes in specular highlight area may provide a dynamic proxy for highlight “coverage,” a cue that has been linked to perceived gloss in prior work (e.g., Marlow et al., 2012; Storrs et al., 2021), although the role of its temporal variation in material perception remains to be fully characterized. However, these analyses do not allow us to determine whether observers relied on any specific cue or combination of cues (e.g., metallicity- vs. transparency-related information) and should therefore be interpreted as illustrating the types of information that may become available through active exploration rather than the precise cues used for this task.

### Illumination structure, individual differences and learning in active material discrimination cases

Even when the material category remained identical, observers’ responses varied substantially across different illumination environments, consistent with prior work demonstrating strong lighting-dependent modulation of material appearance (Kim & Marlow, 2016; Xiao et al., 2014). Our experiments included not only natural illumination maps captured from real-world scenes but also an artificial checker-pattern light field (Figures 2E and Supplementary Figure S2G). Under natural lighting, observers could exploit priors such as the light-from-above assumption (Adams, Graf, & Ernst, 2004; Adams et al., 2016). For example, in one of the natural illumination conditions used in this study (“Sky”), a dominant light source from above (i.e., the sun) was present. Observers may therefore have relied on cues such as increased brightness within the object when viewing the light source through it or preservation of the background color within or near the object boundary, as indicators of glass-like material. Under artificial illumination, performance was reduced relative to that under natural lighting. Nevertheless, observers still exhibited reliable discrimination performance, even though purely static cues would have made the task nearly impossible (Tamura et al., 2018). These results suggest that, regardless of the illumination structure, the dynamic information generated through viewpoint changes and object manipulation provides a primary source of information for resolving material identity. This interpretation further supports the view that active exploration enables observers to compensate for ambiguities introduced by complex illumination conditions.

Quantifying the participants’ movements allowed us to reveal individual differences in how efficiently the observers extracted diagnostic cues during active explorations (Figure 4). In the context of the present study, efficiency refers to exploratory strategies that achieve relatively high discrimination performance without requiring prolonged or redundant exploratory movements. Under traditional passive-viewing paradigms, all observers receive identical retinal inputs, limiting the extent to which individual material perception variability can be expressed. In contrast, active sensing enables substantial performance differences because observers differ not only in their low-level sensory abilities but also in the higher-level strategies that they deploy when selecting and integrating cues (Ernst & Banks, 2002; Maloney & Zhang, 2010; Norman et al., 2016). Because our participants were young adults, the observed differences likely reflect strategic sampling variations rather than age-related sensory declines. However, the origins of these strategy differences remain unclear. Moreover, because exploratory strategies varied across trials, differences in how participants adapted their behavior may have contributed to the observed performance variability. Alongside theoretical proposals that stipulate that material perception emerges from data-driven learning over development and that humans can often generalize to new materials from very small numbers of exemplars, this pattern suggests that both long-term experience and relatively rapid learning processes may shape how efficiently observers discover and exploit diagnostic cues (Fleming, 2017).

### Additional analyses

Although the main analyses focused on translational movements for clarity, we also examined rotational movements to assess whether similar patterns emerged across different modes of exploration (see Supplemental Information; Supplementary Figure S7). Rotational movements showed a pattern broadly consistent with the main analyses based on translational movements. In Experiment 1, the amount of hand rotation increased when the material was most ambiguous, although the correlation between rotational movement and discrimination accuracy was not reach statistically significant. In Experiment 2, head rotations increased under ambiguous material conditions, and the relationship between head rotation and discrimination sensitivity closely mirrored the translational results: a significant positive correlation was observed when hand tracking was disabled, whereas no significant correlation was found when hand tracking was enabled. In Experiment 3, hand rotations again increased for ambiguous materials, but rotational movement did not significantly correlate with discrimination sensitivity regardless of the head-tracking condition. Taken together, these findings suggest that for head movements, translational and rotational measures largely capture similar exploratory behavior. One possible explanation is that both head translation and head rotation generate comparable viewpoint trajectories around the object, effectively producing circular movements of the observer relative to the stimulus. In contrast, hand translation and hand rotation correspond to more distinct action patterns. Translational hand movements tend to involve bringing the object closer or farther away, whereas rotational movements are typically produced through wrist rotations that change object orientation without substantially altering viewing distance. Because these movements generate different classes of visual information, their relationship with discrimination sensitivity may differ. Importantly, however, a common pattern across all the experiments was that the magnitude of exploratory movement—whether translational or rotational—increased when the material was most ambiguous. This finding suggests that perceptual uncertainty reliably elicits active exploratory behavior, which is consistent with the idea that observers increase movement to obtain additional diagnostic information when static cues are insufficient. This pattern supports the idea that exploratory movements are strategically modulated to resolve perceptual ambiguity, even when different movement types generate distinct forms of visual information.

An additional analysis revealed how total exploration duration varied across movement conditions (see Supplemental Information; Supplementary Figure S8). The results revealed a significant main effect of hand availability and an interaction between head and hand movements, with a consistent pattern across both Experiments 2 and 3. When hand movements were allowed, participants tended to spend more time exploring the objects. This likely reflects the additional actions afforded by manual manipulation, such as grasping and repositioning the object, which naturally extend the duration of exploration. Interestingly, allowing both head and hand movements did not substantially increase exploration time relative to the hand-only condition. One possible interpretation is that access to both movement types enabled participants to obtain relevant visual information more efficiently, offsetting the potential increase in exploration time that might otherwise arise from additional degrees of freedom. In contrast, when hand movements were restricted, participants had fewer means to actively sample visual information and therefore tended to make their judgments relatively quickly. These results suggest that exploratory duration is influenced not only by task demands but also by the range of actions available to the observer during active inspection.

We also examined whether exploration duration changed across blocks as participants became more familiar with the task (see Supplementary Figure S9). Exploration duration decreased across blocks in both Experiments 2 and 3, with a significant main effect of block observed in each experiment. This pattern suggests that participants became more efficient over time at identifying informative cues for the task. In addition, a significant interaction between block and hand condition was observed only in Experiment 2, with a steeper decrease in exploration duration when hand movements were available. Despite this effect, the overall pattern of results was largely dominated by the availability of head and hand movements and their interaction, which were consistently observed across both experiments. Together, these results suggest that although exploration became more efficient over time, the structure of the available exploratory actions—whether observers could move their head, their hands, or both—had a stronger influence on exploration behavior than experience-dependent changes did.

To further clarify how participants deployed exploratory movements, we conducted an additional analysis of head position distributions across conditions. Specifically, we computed the mean three-dimensional head position for each trial and compared their spatial distributions between the head-only and head + hand conditions in both Experiments 2 and 3. In Experiment 2 (Supplementary Figure S10A), head position distributions were broadly similar across conditions. However, axis-specific analyses revealed that hand availability modulated head movements along the anteroposterior and vertical axes but not along the lateral axis, suggesting that participants coordinated head movements with manual object manipulation while continuing to rely on lateral viewpoint changes. Notably, head positions were on average biased below the object, indicating a general tendency to observe the stimulus from lower viewpoints. This pattern is consistent with the idea that viewpoint motion alone provides sufficient access to diagnostic information. In contrast, in Experiment 3 (Supplementary Figure S10B), head movements were more spatially extensive in the head-only condition but more constrained in the head + hand condition. Axis-specific analyses further revealed that differences emerged along the vertical and lateral axes but not along the anteroposterior axis, indicating that participants relied less on depth-related head movements and more on object manipulation when manual interaction was available. Consistent with this interpretation, overall head-movement dispersion was reduced when hand movements were available. These results suggest that the relative contribution of head and hand movements depends on how effectively each mode of exploration provides task-relevant information, with observers flexibly reallocating their exploratory behavior accordingly.

### Limitations and directions for future research

One limitation of this research pertains to the range of materials tested. The present study focused exclusively on metal and glass. At first glance, this restriction may appear to limit the generalizability of our conclusions. However, distinguishing metal from glass is among the most challenging problems in the material perception domain because the appearances of both materials depend strongly on the associated environmental illumination conditions and arises from the complex interplay between specular reflection and refraction (Kim & Marlow, 2016; Ohara et al., 2020; Ohara et al., 2022; Tamura et al., 2018, Tamura et al., 2022; Tamura & Nakauchi, 2018). Because these optical properties generate rich context-dependent image structures, metal–glass discrimination provides a useful and well-controlled case for examining how active exploration contributes to material perception. Nevertheless, the extent to which the context-dependent sampling strategies observed here can be generalized to other material classes—such as wood, fabric, plastic, or liquid—remains unclear. Future work should expand the stimulus space to include materials with diverse reflectance, refractive, and subsurface scattering properties to identify the boundary conditions and general domain principles of active material perception. Large-scale analyses of material judgments indicate that diverse materials can be described by a compact set of shared perceptual dimensions (Schmidt, Hebart, Schmid, & Fleming, 2025), suggesting that some of the cues leveraged in metal–glass discrimination tasks may also support judgments of other material classes. Nevertheless, direct tests will be required to determine the breadth of this generalization.

Moreover, the present study focused on rigid objects, whereas many real-world materials do not maintain fixed shapes, and how observers actively interact with such materials remains largely unknown. Even nominally solid objects can deform under active manipulation, revealing cues related to elasticity or stiffness (Bi, Jin, Nienborg, & Xiao, 2019; Paulun et al., 2017; Paulun & Fleming, 2020; Schmidt, Paulun, van Assen, & Fleming, 2017). In addition, observers often form predictions about the likely material properties of an object before interacting with it (Alley, Schmid, & Doerschner, 2020; Kaiser, Stecher, & Doerschner, 2023), and some materials may even fracture or break upon contact (Schmid & Doerschner, 2018). When observers interact with such deformable stimuli, exploratory behavior may reveal not only differences in visual appearance but also strategies for actively probing how the material responds to manipulation. Such behaviors are consistent with the broader view that perception of natural behavior involves active sampling of task-relevant information through self-generated actions (Goettker et al., 2025). In this sense, material perception may involve not only selecting informative viewpoints but also adopting higher-level exploratory strategies that determine how the viewing geometry is altered—either through viewpoint motion or through object manipulation—to reveal diagnostic information. By demonstrating how active exploration supports the discrimination of rigid materials, the present findings provide a foundation for extending active-sensing approaches to broader classes of deformable and fragile materials.

A second limitation concerns the use of virtual reality. Although VR allowed us to experimentally dissociate head and hand movements—two sources of information that are tightly coupled in natural behaviors—this very dissociation is artificial in real-world settings. Moreover, the ability to manipulate these exploratory modes independently is a key strength of VR, enabling us to demonstrate that observers flexibly reweight their sampling strategies depending on which exploratory mode provides more diagnostic information. It remains an open question whether the same context-dependent strategy switching emerges when head and hand movements are intrinsically coupled in the physical world. A related question concerns the ecological validity of providing simultaneous reference objects, as done in Experiment 2. Although real environments contain many materials, situations requiring observers to directly compare multiple surfaces with high reflectance or transparency levels may be relatively rare. In contrast, scenarios that more closely resemble Experiment 3, where a single target object must be judged among a heterogeneous set of background materials, may capture the material identification problems that are typically faced in everyday vision settings more effectively.

Furthermore, real-world material perception is an inherently multisensory process (Fujisaki, Goda, Motoyoshi, Komatsu, & Nishida, 2014; Spence, 2020). The haptic feedback obtained when interacting with an object (e.g., surface compliance, roughness, or temperature (Dövencioǧlu, Üstün, Doerschner, & Drewing, 2022; Jones & Ho, 2025; Okamoto, Nagano, & Yamada, 2013)) and physical cues such as weight or thermal conductivity can substantially influence material judgments. Although our VR setup included minimal vibrotactile feedback when the virtual hand contacted the object to enhance the degree of immersion, it did not provide any diagnostic haptic information that was relevant to the material properties. Thus the present study effectively isolated the visual component and demonstrated that observers deploy context-dependent strategies based primarily on visual explorations. This framework provides a foundation for future researchers to investigate how active visual sampling can be integrated with richer haptic and proprioceptive signals during naturalistic material recognition processes.

A third limitation concerns the acquisition of active sampling strategies. Our results showed that individual differences in how participants explored the stimulus—such as pulling the object, approaching it, or viewing it from below or above—were systematically related to material discrimination sensitivity and changes in performance across trials (Figure 4). This suggests that the visual system may benefit from efficient exploration strategies, yet how such strategies are acquired remains unknown. Do observers possess innate preferences for certain visual or motion cues, or are these strategies learned through experience? Notably, even within the few hundred trials of our experiment, some participants appeared to implicitly (or explicitly) discover more efficient motion patterns and subsequently increased their discrimination sensitivities. This raises the possibility that strategy discovery and refinement occur on relatively short timescales. One promising extension of the present study is to analyze movement trajectories and viewpoint sequences in greater detail—linking motion features and video-based appearance cues to trial-by-trial decision outcomes. Computational modeling may also help identify which cues an ideal observer would exploit and whether human observers converge on similar strategies (Cheeseman et al., 2025; Liao et al., 2023; Morimoto et al., 2025; Prokott et al., 2021; Prokott & Fleming, 2022; Storrs et al., 2021; Tamura et al., 2022; van Assen et al., 2020), thereby clarifying which aspects of active material perception reflect learned behaviors versus the inherent constraints imposed on the visual system. In addition, it will be important to directly identify which dynamic visual cues are generated during active exploration and how these cues are selectively utilized for material judgments.

## Conclusions

Our findings suggest that human observers flexibly adapt their active sampling strategies to viewing-context constraints and that such strategies are associated with increasing material discrimination sensitivity and changes in performance over time. These results are consistent with a theoretical framework in which material perception is understood not solely as a passive static image decoding procedure but rather as an adaptive process influenced by task demands and exploratory behaviors, highlighting aspects of material perception that may be difficult to capture under traditional passive-viewing paradigms.

## Supplementary Material

Supplement 1

Supplement 2

Supplement 3

## Supplementary material

Supplementary Movie S1. Representative first-person views from a single trial. A metal stimulus (metallicity = 1.0). The illumination condition was Sky, and the object geometry was Bumpy. The video corresponds to the left-eye view from the binocular recordings obtained via the head-mounted display. The viewing conditions change over time: Static View (0–6 s), Hand Only (6–18 s), Head Only (18–32 s), and Head & Hand (32 s onward).

Supplementary Movie S2. Representative first-person views from a single trial. A glass stimulus (metallicity = 0.0). The illumination condition was Sky, and the object geometry was Bumpy. The video corresponds to the left-eye view from the binocular recordings obtained via the head-mounted display. The viewing conditions change over time: Static View (0–6 s), Hand Only (6–18 s), Head Only (18–32 s), and Head & Hand (32 s onward).
