# Supplementary material for: Material discrimination relies on context-dependent active sensing strategies
Source: J Vis. 2026 Jun 8;26(6):4. doi: 10.1167/jov.26.6.4 (PMC13263983; doi:10.1167/jov.26.6.4)
Supplement: Supplement 1 [file jovi-26-6-4_s001.pdf]

Supplemental Information

Material discrimination relies on context-dependent active sensing strategies

Ryu Nomachi[†], Hideki Tamura[†,*], Kevin A. Helgeland, Takuma Morimoto, Shigeki Nakauchi, Tetsuto Minami

1. Renderings

In the physically based rendering (PBR) model implemented in HDRP, the metallic parameter m determines both the normal-incidence reflectance $\mathrm{F_0}$ of the surface and the diffuse reflection component $\mathrm{C_{diffuse}}$. Specifically, using the baseline dielectric reflectance $\mathrm{k_{dielectric}} \approx 0.04$ and the specified albedo color $\mathrm{C_{albedo}}$, these quantities are computed as follows:

$$\mathrm{F_0} = (1 - \mathrm{m}) \cdot 0.04 + \mathrm{m} \cdot \mathrm{C_{albedo}}$$

$$\mathrm{C_{diffuse}} = \mathrm{C_{albedo}} \cdot (1 - \mathrm{m})$$

Under the condition in which the surface type was set to transparent, a value of $\mathrm{m} = 0$ defines the material as a dielectric. In this case, most of the incident light is transmitted through and refracted within the object, producing a glass-like appearance. As m increases, the transmission component decreases due to energy conservation, while the reflected component becomes stronger

and is increasingly tinted by C_{albedo}. When $m = 1$, both diffuse reflection and transmission vanish, and surface reflection becomes dominant, resulting in the appearance of a metallic material. Through this manipulation of the metallic parameter, the same shader program supports a continuous transition in material appearance from a transmissive dielectric to a reflective conductor. Further technical details of the PBR framework can be found in the following references:

- Burley (2012), Physically-Based Shading at Disney
  https://media.disneyanimation.com/uploads/production/publication_asset/48/asset/s2012_pbs_disney_brdf_notes_v3.pdf

- Unity Technologies documentation:
  https://docs.unity3d.com/6000.3/Documentation/Manual/StandardShaderMetallicVsSpecular.html

2. Results of a matching experiment

To validate the perceptual plausibility of the material stimuli rendered in Unity, we conducted a material-matching experiment using these stimuli. During the experiment, participants' head positions were stabilized using a chin rest. The target stimulus consisted of a three-dimensional rendered object with one of five levels of metallicity (0, 0.25, 0.5, 0.75, 1.0), identical to those used in the main experiment, and was presented in front of the participant. The reference stimuli consisted of nine two-dimensional images rendered using physically based rendering, following Tamura et al. (2018), with refractive indices of 1.33 (reflectance $R = 0.04$, transmittance $T = 0.96$), 1.5, 1.7, 2.42, 5, 10, 20, 50, and infinity ($R = 1.0$, $T = 0.0$). One reference image was presented at a time alongside the target.

Participants were allowed to switch between the reference images using button presses and were instructed to select the reference stimulus that most closely matched the material appearance of the target from the nine options. The experimental conditions consisted of all combinations of five levels of metallicity, two object geometries (bumpy and smooth, as in the main experiment), and five illumination conditions (identical to those used in Tamura et al., 2018, but different from those in the main experiment). Each participant completed 100 trials (5 metallicity levels × 2 geometries × 5 illumination conditions × 2 repetitions).

The distribution of matched refractive indices across all participants is shown in Figure S1. Overall, the results showed that higher levels of metallicity were associated with matches to stimuli with higher refractive indices.

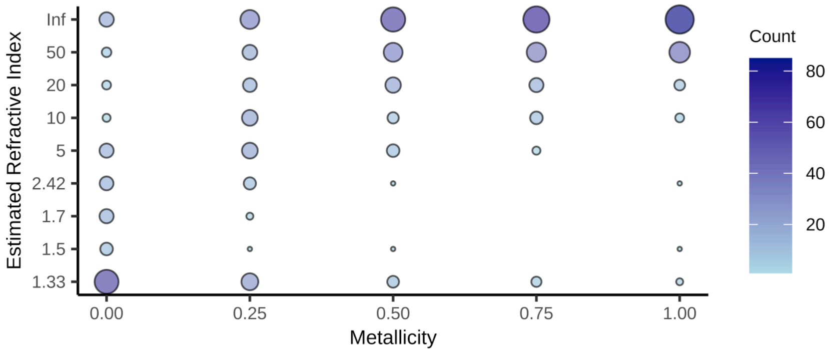

Figure S1. Results of a matching experiment showing the correspondence between the metallicity parameter and the refractive index obtained from the physically based rendering procedure (n = 7).

3. Stimuli details and responses in Experiment 3

To increase the variability of the stimuli, two object geometries (Figure S2A) and multiple illumination conditions were employed. Specifically, six illumination environments were used in Experiment 1 (Figure S2B), and four illumination environments were used in Experiments 2 and 3 (Figure S2C). The combinations of the seven metallicity levels and the four illumination conditions used in Experiment 2 are shown in Figure S2D.

We also present the response data from Experiment 3 (corresponding to Figures 2C–2F). Compared to Experiment 2, the response functions in Experiment 3 were overall more gradual. Nevertheless, the key patterns were largely consistent across Experiments 2 and 3: responses under natural illumination differed from those under artificial illumination (Figure S2G), and the proportion of metal responses was higher for bumpy objects than for smooth objects (Figure S2H).

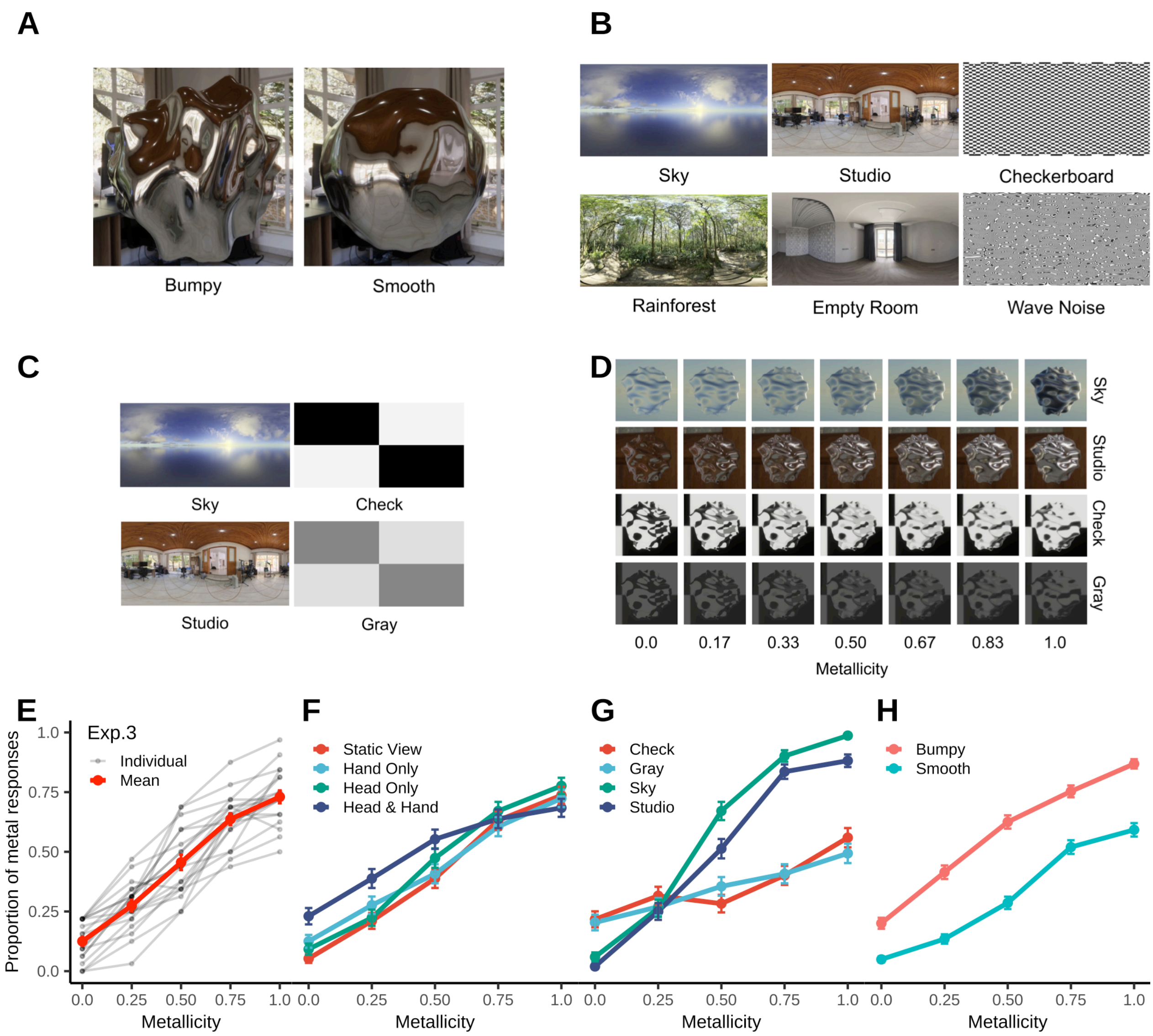

A
Bumpy
Smooth
B
Sky
Studio
Checkerboard
Rainforest
Empty Room
Wave Noise
C
Sky
Check
Studio
Gray
D
Sky
Studio
Check
Gray
0.0
0.17
0.33
0.50
0.67
0.83
1.0
Metallicity
E
Exp.3
Individual
Mean
Proportion of metal responses
Metallicity
F
Static View
Hand Only
Head Only
Head & Hand
Metallicity
G
Check
Gray
Sky
Studio
Metallicity
H
Bumpy
Smooth
Metallicity

Figure S2. (A) Examples of stimulus shapes: a highly bumpy object (left) and a relatively smooth object (right). (B) Examples of the six illumination environments used in Experiment 1. Columns from left to right: outdoor, indoor, and artificial environments. (C) Examples of the four illumination environments used in Experiments 2 and 3. From left to right, the columns denote natural and artificial environments. (D) Examples of the stimuli used in Experiments 2 and 3, illustrating the material appearance manipulations made along the metallicity parameter from 0.0 (left) to 1.0 (right). (E–H) Metal response data obtained in Experiment 3: overall and individual results (E), plotted separately by experimental condition (F), illumination environment (G), and object shape (H).

4. Categorization of exploration duration

Participants' exploratory behavior was categorized into four types, and the time spent in each category was quantified (Figures S3A and S3B). Because head movements were tracked with six degrees of freedom, participants could change their viewpoint through both translation and rotation, allowing them to observe the object from different elevations even when object manipulation was disabled. Behavioral labels were assigned on a frame-by-frame basis according to predefined criteria.

"Pulling" was defined as instances in which the stimulus–head distance was <= 40 cm and the stimulus moved farther from its initial position than the head. "Approaching" was defined as instances in which the stimulus–head distance was <= 40 cm and the head moved farther than the stimulus. "Looking up" was defined as instances in which the stimulus was positioned more than 5 cm above eye level, whereas "Looking down" was defined as instances in which the head was more than 5 cm above the stimulus.

Note that these behavioral categories reflect time spent under specific criteria and do not directly correspond to the average head position within a trial. For example, although head positions were often located below the object, the proportion of time classified as "looking up" was not necessarily dominant (see Figure S10).

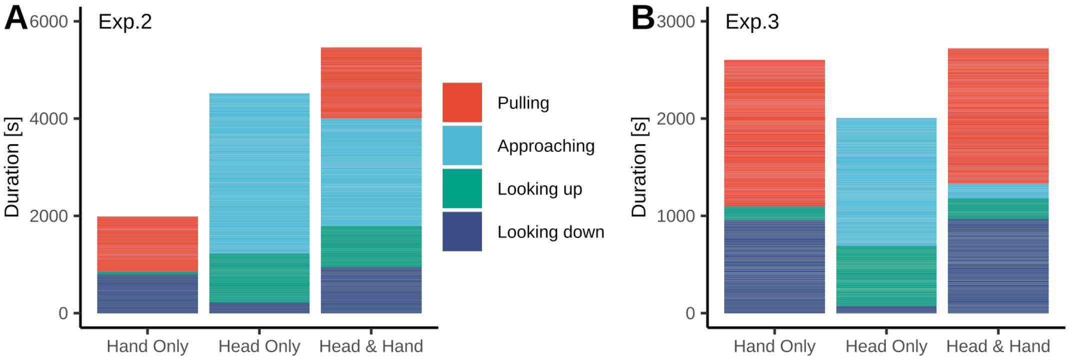

Figure S3. (A) Total exploration time required for all participants in Experiment 2. (B) Total exploration time required for all participants in Experiment 3.

5. Example trajectories of head and hand movements

Based on the cluster analysis of individual differences in exploration time in the head + hand condition of Experiment 2 (Figure 4A), we selected one representative participant from each cluster under the same condition. We then visualized the head and hand trajectories of these three participants during a representative trial under a specific stimulus condition (material: metallicity = 0.5, shape: bumpy, and illumination: gray; Figure S4). Participant 13 (Cluster 1) showed extensive head movements with relatively limited hand movements, suggesting a head-dominant strategy (Figure S4A). In contrast, Participant 20 (Cluster 2) actively moved both the head and the hands, reflecting a combined exploration strategy (Figure S4B). Participant 11 (Cluster 3) exhibited minimal movement of both the head and the hands, indicating a low-exploration strategy (Figure S4C). These examples illustrate qualitatively distinct patterns of exploratory behavior across individuals.

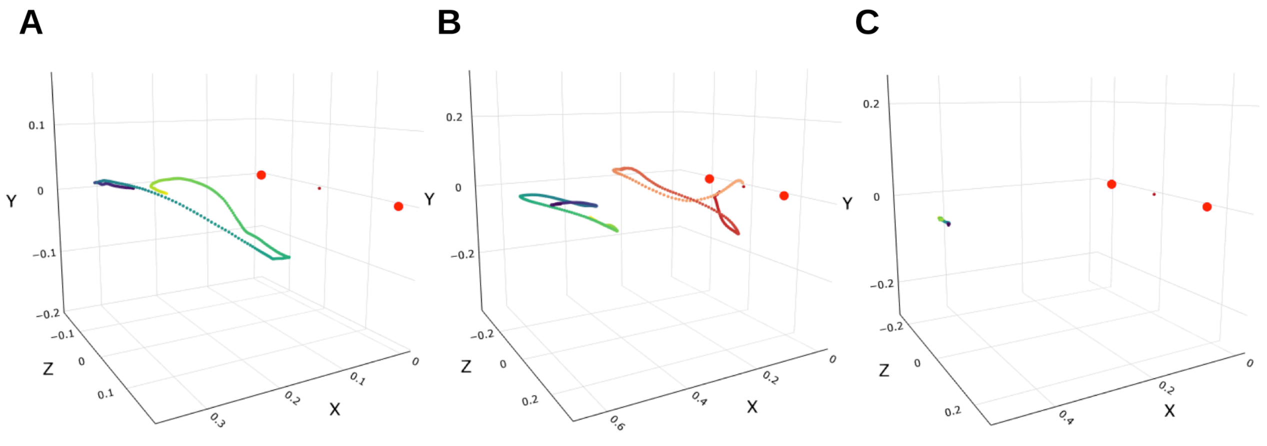

Figure S4. Example trajectories of head and hand movements during a single trial. Coordinates are defined in an observer-centered (egocentric) reference frame: the X-axis represents depth (anterior–posterior; negative values indicate movement toward the stimulus, i.e., forward relative to the observer), the Y-axis represents the vertical position (superior–inferior), and the Z-axis represents the horizontal position (lateral; left–right). The bluish trajectories on the left side of each panel correspond to head movements, whereas the reddish trajectories on the right side correspond to hand movements. In addition, the two red dots on the right side indicate the positions of the reference stimuli in Experiment 2. Panel (A) shows data from Participant #13 (Cluster 1), (B) from Participant #20 (Cluster 2), and (C) from Participant #11 (Cluster 3).

6. Detection of “behind-the-object” exploration strategy

To assess whether participants adopted a strategy of placing their hand behind the object to inspect its transparency, we conducted an additional analysis using the data from Experiment 3, in which hand positions were recorded.

We defined candidate events for this strategy based on the relative spatial configuration of the camera (HMD), the object, and the hand. Specifically, a trial was flagged when all of the following criteria were met: (i) the angle between the camera-to-object vector and the camera-to-hand vector was less than 15°, (ii) the hand was located behind the object relative to the camera (i.e., farther from the camera than the object along the viewing direction), and (iii) the hand was not grasping the object. In addition, these conditions had to be sustained for at least 1 second within a trial.

Applying these criteria, such events were detected in 32 trials out of all trials in which manual interaction was available (80 trials × 19 participants), corresponding to approximately 2% of trials. This finding indicates that, although this strategy was physically possible, it was rarely adopted by participants. These results further support the conclusion that participants did not systematically rely on a “behind-the-object” inspection strategy.

7. Illustration of dynamic image features during active exploration

To examine the types of dynamic image information generated by object manipulation, we conducted additional analyses using data from Experiment 3, in which both hand movements and viewing behavior were recorded. For a representative trial in which the stimulus was judged as metal, we extracted three time-resolved measures: (i) the ratio of specular highlight area relative to the object area, (ii) the divergence of optic flow within the stimulus region, and (iii) hand translation (Figure S5). These measures were computed frame-by-frame to characterize the temporal relationship between object manipulation and image changes.

To quantify this relationship across trials, we computed the temporal variability (standard deviation) of highlight area for each trial and related it to a hand-movement index, defined as total hand translation normalized by exploration time (Figure S6). These measures were selected to capture complementary aspects of dynamic visual information relevant to gloss perception. In particular, optic flow divergence has been proposed as a diagnostic cue for gloss (Doerschner et al., 2011), whereas changes in specular highlight area provide a proxy for the proportion of the surface covered by specular reflections (“coverage”), a cue known to be related to perceived gloss (e.g., Marlow et al., 2012; Storrs et al., 2021). Although the temporal variability of highlight area has not been explicitly formalized as a standalone metric, it is broadly consistent with proposals that coverage-related changes contribute to motion-based gloss perception.

Specular highlight area was estimated using a simple intensity-based criterion. RGB images were converted to a luminance-like measure (Y) assuming sRGB encoding, and pixels with Y >= 200 (on an 8-bit scale) were classified as belonging to the highlight region. Because the display was not photometrically calibrated, this measure does not correspond to absolute luminance but instead provides a relative index of high-intensity image regions. This threshold-based approach thus serves as a coarse but consistent proxy for specular highlights.

Optical flow was computed using the Lucas–Kanade method implemented in OpenCV (cv2.calcOpticalFlowPyrLK) in Python. For each pair of consecutive frames, a two-dimensional optical flow field was estimated within the stimulus region. Following Doerschner et al. (2011), we quantified the divergence of the optical flow field. This measure captures the local expansion or contraction of motion signals in the image. To obtain a direction-invariant index of motion magnitude, we computed the absolute value of divergence at each pixel and averaged it across the stimulus region, yielding the mean absolute divergence for each frame.

In the representative trial (Figure S5), changes in specular highlight area and optic flow divergence tended to co-occur with hand movements, indicating that object manipulation can induce structured temporal changes in image features. Across trials, the variability in highlight area was correlated with the hand movement index (Figure S6A: $r = 0.85$, $p < .001$), but not with the head movement index (Figure S6B: $r = 0.38$, $p = .104$), suggesting that larger hand movements are

associated with greater temporal changes in highlight structure. These results illustrate that object manipulation can generate dynamic visual information, such as changes in specular flow. However, these analyses do not establish that observers relied on any specific cue or combination of cues and should be interpreted as illustrating the types of information that may become available during active exploration.

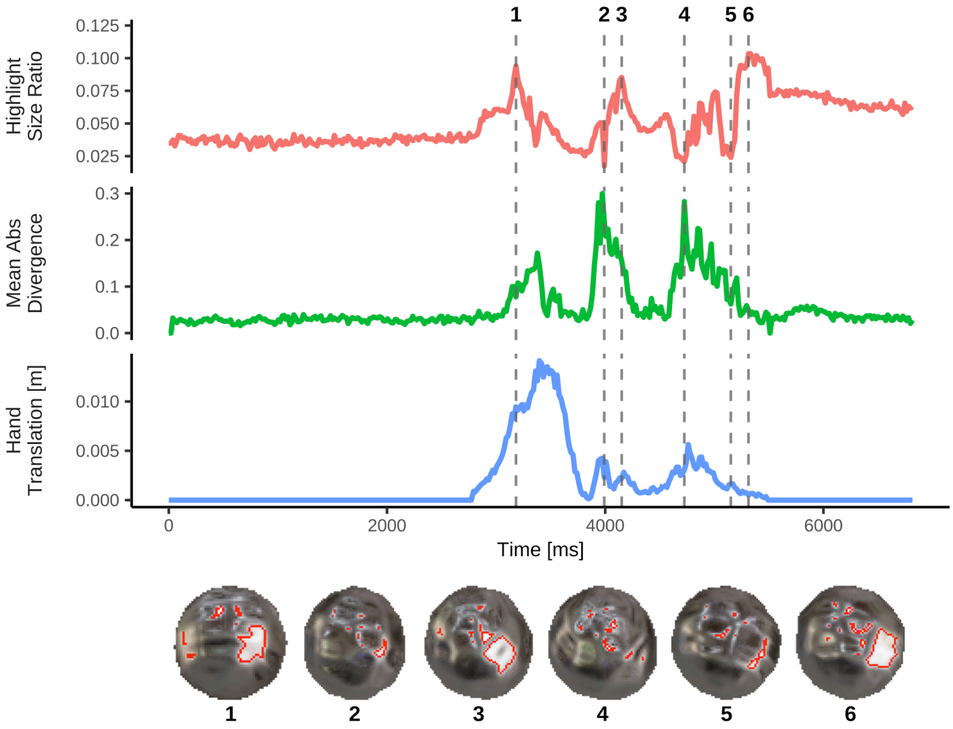

Figure S5. Temporal evolution of feature measures in a representative trial. The top panel shows the proportion of the specular highlight area, the middle panel shows the absolute divergence of optic flow, and the bottom panel shows hand translation. The snapshots at the bottom illustrate the specular highlight regions (outlined in red) at six selected time points corresponding to moments when the specular highlight area ratio was relatively high or low.

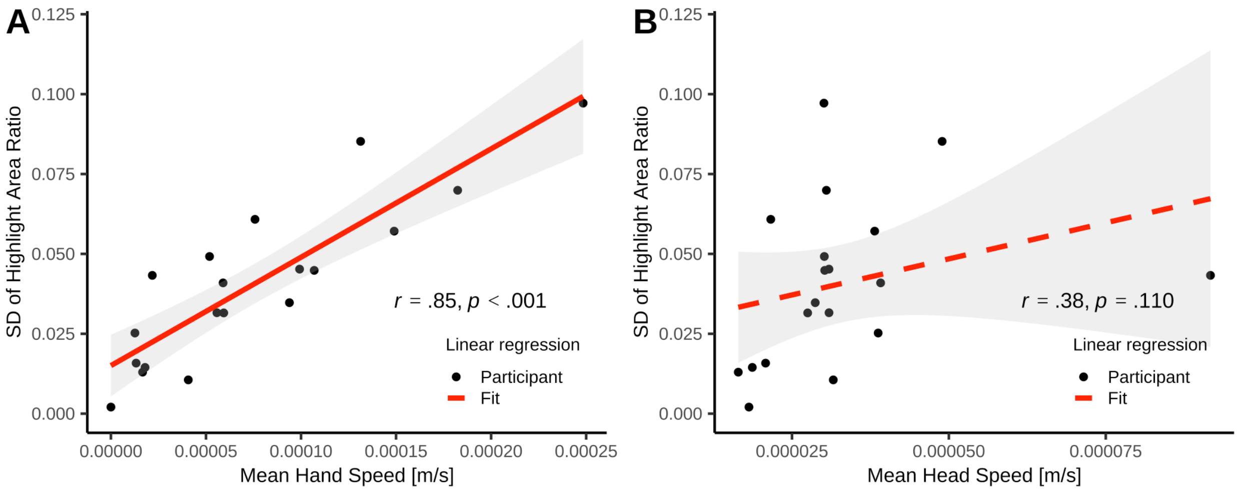

Figure S6. Relationship between movement speed and temporal variability (SD) in the specular highlight area. (A) In Experiment 3, hand movement speed (defined as total hand translation divided by exploration time) and the temporal variability of specular highlight area were computed for trials in which hand movement was allowed and then averaged for each participant. (B) Same as (A), but with hand movement speed replaced by head movement speed for trials in which head movement was allowed.

8. Rotational movements

Across all experiments, rotational movements increased when the material was most ambiguous, consistent with the pattern observed for translational movements. In Experiment 1, the amount of hand rotation was greatest for ambiguous materials, although the correlation between rotation and discrimination accuracy was not statistically significant ($r = .42,\ p = .086$). In Experiment 2, head rotation also increased under material ambiguity. The relationship between head rotation and sensitivity closely mirrored the translational results: a significant positive correlation was observed when hand tracking was disabled ($r = .70,\ p < .001$), whereas no significant correlation was found when hand tracking was enabled ($r = .26,\ p = .274$). In Experiment 3, hand rotation again increased for ambiguous materials, but hand rotational movement was not significantly correlated with the sensitivity regardless of the head-tracking condition (head-on: $r = .15,\ p = .573$; head-off: $r = .29,\ p = .247$).

Overall, head translation and head rotation produced similar results (Experiment 2), possibly because both movements generate comparable viewpoint trajectories around the object. In contrast, hand translation and hand rotation correspond to more distinct action patterns and may therefore provide different visual information. Nevertheless, a consistent finding across all experiments was that exploratory movement increased when the material was most ambiguous, suggesting that perceptual uncertainty reliably elicits active exploratory behavior.

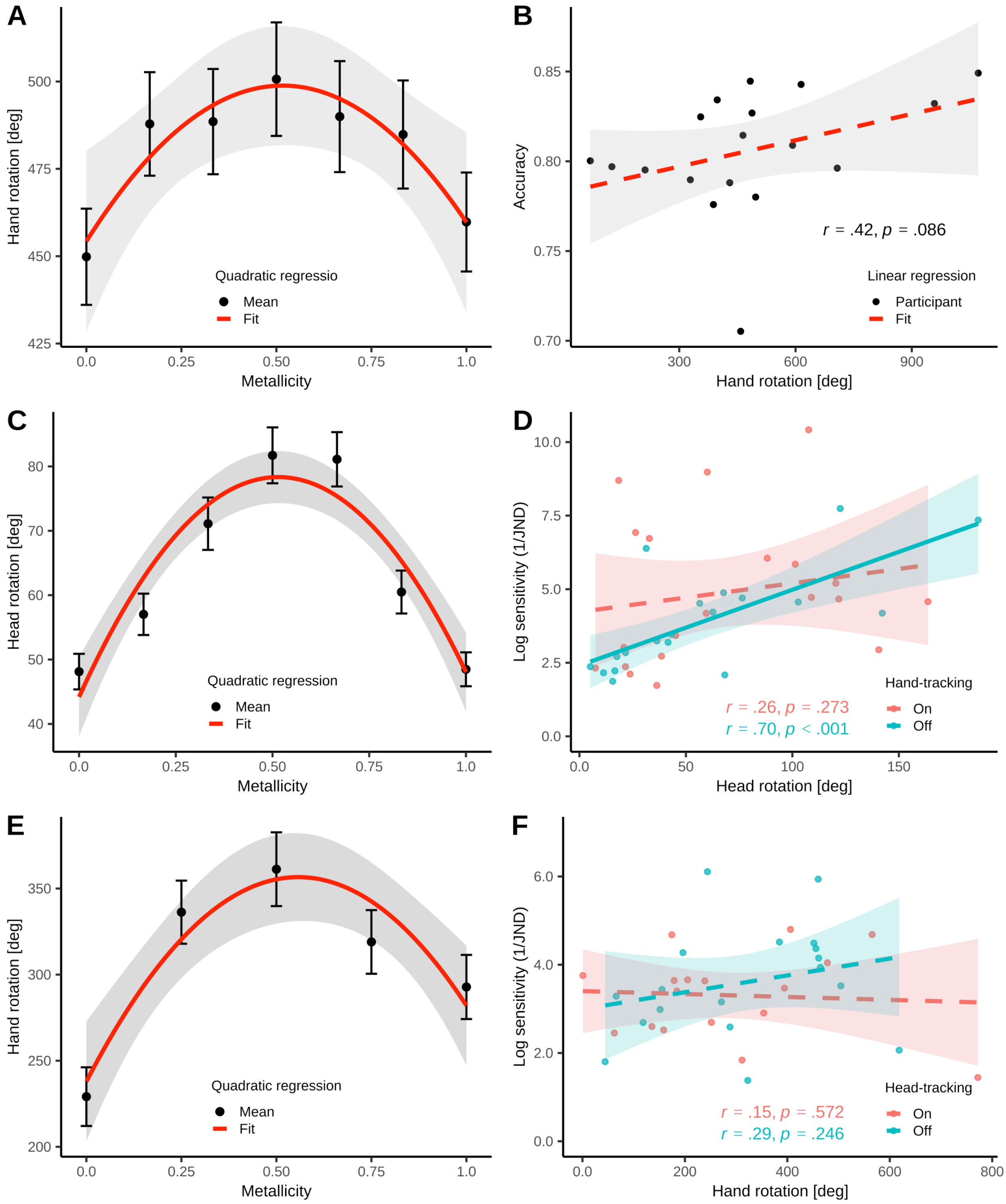
A
Hand rotation [deg]
500
475
450
425
Quadratic regressio
Mean
Fit
0.0
0.25
0.50
0.75
1.0
Metallicity
B
Accuracy
0.85
0.80
0.75
0.70
r = .42, p = .086
Linear regression
Participant
Fit
300
600
900
Hand rotation [deg]
C
Head rotation [deg]
80
70
60
50
40
Quadratic regression
Mean
Fit
Metallicity
D
Log sensitivity (1/JND)
10.0
7.5
5.0
2.5
0.0
Hand-tracking
r = .26, p = .273
r = .70, p < .001
On
Off
0.0
50
100
150
Head rotation [deg]
E
Hand rotation [deg]
350
300
250
200
Quadratic regression
Mean
Fit
Metallicity
F
Log sensitivity (1/JND)
6.0
4.0
2.0
0.0
Head-tracking
r = .15, p = .572
r = .29, p = .246
On
Off
0.0
200
400
600
800
Hand rotation [deg]

Figure S7. (A) Relationship between metallicity and rotation and between rotation and performance. Using rotation instead of translation, panels (A–B) follow the same format as Figures 1G–H, panels (C–D) as Figures 2H–I, and panels (E–F) as Figures 3D–E.

9. Total exploration duration

To further characterize exploratory behavior, we analyzed the total exploration duration in Experiments 2 and 3. For each trial, total exploration duration was defined as the elapsed time from stimulus onset to the participant's response. Because the distribution of exploration duration was positively skewed, the values were log-transformed prior to statistical analysis. For each experiment, we fitted a linear mixed-effects model with head-tracking condition (Head), hand-tracking condition (Hand), and their interaction as fixed effects, and participant as a random intercept: $\log(\mathrm{Time}) \sim \mathrm{Head} * \mathrm{Hand} + (1|\mathrm{Participant})$, where Time denotes the total exploration duration in each trial. The purpose of this analysis was to test whether the availability of head- and hand-based exploratory actions influenced the overall time spent exploring the stimulus before making a judgment.

The analysis of total exploration duration revealed the same overall statistical pattern in both Experiment 2 and Experiment 3 (Figure S8). In both experiments, there was a significant main effect of hand availability (Exp. 2: $F(1,57.00) = 77.56, p < .001, \eta_p^2 = .576$; Exp. 3: $F(1,54.00) = 61.43, p < .001, \eta_p^2 = .532$) and a significant Head × Hand interaction (Exp. 2: $F(1,57.00) = 8.94, p = .004, \eta_p^2 = .136$; Exp. 3: $F(1,54.00) = 15.20, p < .001, \eta_p^2 = .220$), indicating that exploration duration varied systematically depending on which movement types were available. Follow-up pairwise comparisons further showed that, regardless of whether head movements were available, exploration duration was longer when hand movements were available than when they

were unavailable (Exp. 2: Head on: $t(57.00) = 4.11$, 95% CI [0.09, 0.44], $p < .001$, $d = .54$; Head off: $t(57.00) = 8.34$, 95% CI [0.36, 0.72], $p < .001$, $d = 1.10$; Exp. 3: Head on: $t(54.00) = 2.79$, 95% CI [0.00, 0.50], $p = .044$, $d = .38$; Head off: $t(54.00) = 8.30$, 95% CI [0.50, 0.99], $p < .001$, $d = 1.12$).

This pattern likely reflects the additional actions afforded by manual manipulation, such as grasping, repositioning, and rotating the object, which naturally extended the duration of exploration. In contrast, when hand movements were unavailable, participants had fewer opportunities to actively sample visual information and therefore tended to make their judgments more quickly. Interestingly, allowing both head and hand movements simultaneously did not markedly increase exploration time beyond the level observed in the hand-only condition. Thus, although the availability of both movement types did not shorten total exploration duration, it also did not substantially prolong it beyond the time required for manual exploration alone. One possible interpretation is that when both movement types were available, participants could coordinate them to sample relevant visual information more efficiently, partially offsetting the increase in duration expected from the availability of additional degrees of freedom. Taken together, these results suggest that total exploration duration was shaped not only by perceptual demands but also by the range of actions available to the observer and that this pattern was reproducible across Experiments 2 and 3.

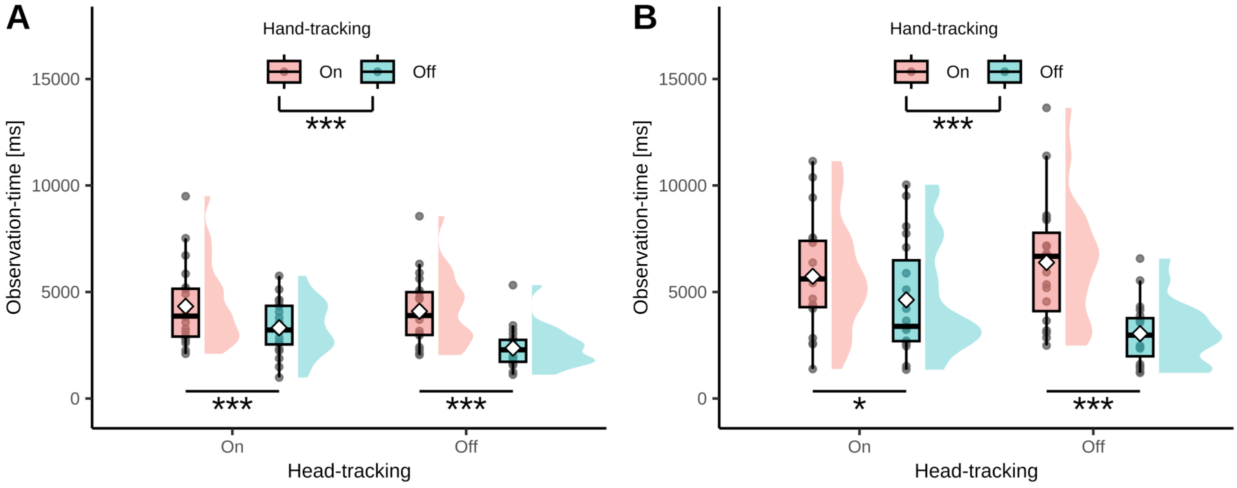

Figure S8. Total exploration duration across movement conditions in Experiments 2 (A) and 3 (B). The mean total exploration duration is shown for each combination of head-tracking and hand-tracking conditions. The format was the same as that in Figure 2G.

10. Total exploration duration across blocks

To examine potential learning effects, we analyzed whether total exploration duration changed across experimental blocks in Experiments 2 and 3. For each trial, exploration duration was defined as the elapsed time from stimulus onset to the participant's response. Because the distribution of exploration duration was positively skewed, the values were log-transformed prior to statistical analysis. We fitted a linear mixed-effects model including head-tracking condition (Head), hand-tracking condition (Hand), block number (Block), and their interactions as fixed effects, with participant as a random intercept: $\log(\text{Time}) \sim \text{Head} * \text{Hand} * \text{Block} + (1|\text{Participant})$. This analysis tested whether exploration duration systematically changed across blocks while accounting for the availability of head- and hand-based exploratory movements.

In both experiments, there was a significant main effect of the block number, with exploration duration decreasing as the experiment progressed (Exp. 2: $F(1,293.00) = 25.36, p < .001, \eta_p^2 = .080$; Exp. 3: $F(1,278.00) = 21.75, p < .001, \eta_p^2 = .073$). This pattern may reflect a learning effect, whereby participants became more efficient at identifying which cues were most informative for the task, leading to shorter exploration times. In addition, a significant interaction between block and hand condition was observed only in Experiment 2 ($F(1,293.00) = 3.98, p = .047, \eta_p^2 = .013$). Exploration time decreased more steeply when hand movements were available compared to when they were not (Figure S9A). One possible interpretation is that, in Experiment 2, cues derived from

head movements (i.e., viewpoint changes) were sufficiently informative, such that manual exploration became relatively less efficient. As a result, even when hand movements were available, participants may have reduced the time spent on manual exploration.

All other effects of hand, head, and their interaction were consistent with the pattern observed in Figure S8 in both experiments.

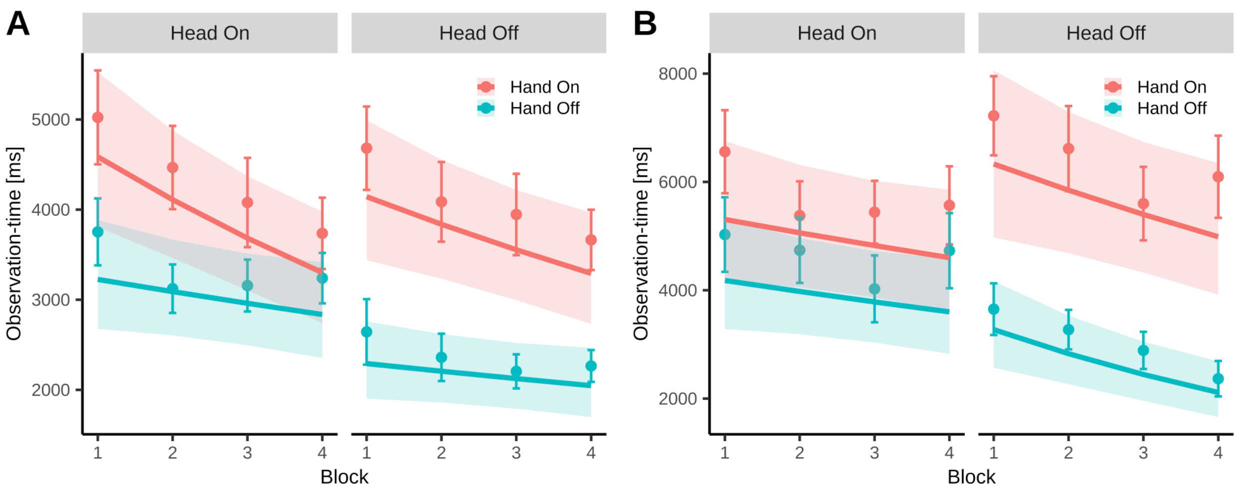

Figure S9. Changes in observation time across blocks. The x-axis represents the block number, and the y-axis represents the observation time per trial. The circles and error bars indicate the mean and standard error, respectively, and the shaded regions represent 95% confidence intervals. (A) Experiment 2. (B) Experiment 3.

11. Head position distributions

To characterize global movement patterns, we analyzed the spatial distribution of head positions across all trials in Experiments 2 and 3. For each trial, the mean three-dimensional head position was computed and plotted as a single point in space (Figure S10). In Experiment 2 (Figure S10A), the distributions of head positions were broadly spread in both the head-only condition (blue) and the head + hand condition (orange), with substantial overlap between conditions. In contrast, in Experiment 3 (Figure S10B), head positions were widely distributed in the head-only condition but were more spatially constrained in the head + hand condition.

To statistically assess these differences, we conducted separate linear mixed-effects models for each spatial dimension (x, y, and z), with hand availability as a fixed effect and participant as a random intercept ($\mathrm{Head}_{\mathrm{position}} \sim \mathrm{Hand} + (1|\mathrm{Participant})$). After Bonferroni correction, significant effects were observed along the x-axis (anteroposterior direction: $\mathrm{F}(1{,}4459.00) = 164.21, \mathrm{p} < .001, \eta_{\mathrm{p}}^{2} = .036$) and y-axis (vertical direction: $\mathrm{F}(1{,}4459.00) = 44.72, \mathrm{p} < .001, \eta_{\mathrm{p}}^{2} = .010$) but not along the z-axis (lateral direction: `r $\mathrm{F}(1{,}4459.00) = 2.00, \mathrm{p} = .473, \eta_{\mathrm{p}}^{2} = .000$) in Experiment 2. These results indicate that the availability of hand movements modulated head motion primarily along the anteroposterior and vertical axes, suggesting that participants moved their heads while simultaneously pulling the stimulus closer or pushing it away during object manipulation. In contrast, the absence of a difference along the lateral axis suggests that horizontal head movements

were comparable regardless of hand availability, implying that lateral viewpoint changes served as an effective strategy for acquiring diagnostic visual information in Experiment 2. Notably, head positions were predominantly located below the object (Y < 0; 75.7% overall, Head Only: 75.5%, Head & Hand: 75.9%), confirming a strong tendency to observe the object from lower viewpoints.

In Experiment 3, no significant difference was observed along the anteroposterior axis (x-axis: $F(1,1500.00) = 2.75, p = .293, \eta_p^2 = .002$), whereas significant differences were found along the vertical and lateral axes (y-axis: $F(1,1500.00) = 52.44, p < .001, \eta_p^2 = .034$; z-axis: $F(1,1500.00) = 591.01, p < .001, \eta_p^2 = .283$). These results suggest that, in Experiment 3—where object manipulation was a more effective strategy—participants did not strongly rely on head movements along the depth axis, regardless of whether hand movements were available. Furthermore, when both head and hand movements were available, participants appeared to rely more heavily on object manipulation than on head movements. A similar tendency was observed in Experiment 3, with head positions predominantly located below the object (Y < 0; 74.0% overall, Head Only: 78.6%, Head & Hand: 71.2%).

In addition, we quantified the overall spatial dispersion of head movements using a composite measure defined as $\text{Dispersion} = \sqrt{SD_x^2 + SD_y^2 + SD_z^2}$, which captures the extent of movement in three-dimensional space. A linear mixed-effects model ($\text{Dispersion} \sim \text{Hand} + (1|\text{Participant})$) revealed that head movement dispersion was significantly greater in the head-only condition than in

the head + hand condition (Exp. 2: $F(1,4459.00) = 37.30, p < .001, \eta_p^2 = .008$; Exp. 3: $F(1,1500.00) = 482.04, p < .001, \eta_p^2 = .243$).

These results suggest that when manual interaction was available, participants could obtain relevant information without extensively moving their heads, likely by reducing viewing distance and accessing informative viewpoints (e.g., from above or below) through hand-based object manipulation.

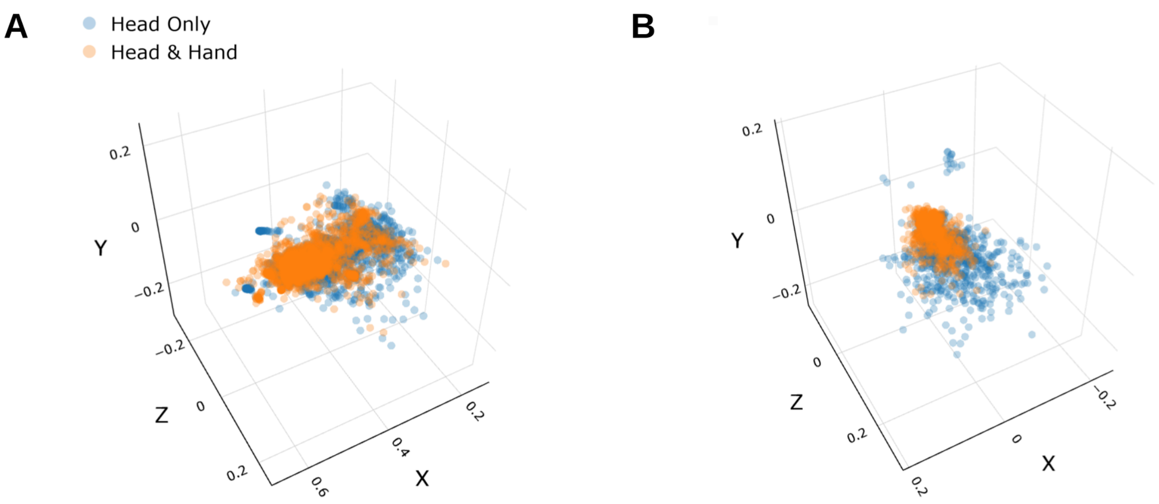

Figure S10. Distribution of head positions. Each point represents the mean head position within a single trial, with all trials across participants plotted. Blue indicates the head-only condition, and orange indicates the head+hand condition. Coordinates are defined in an observer-centered (egocentric) reference frame: the X-axis represents depth (anterior–posterior; negative values indicate movement toward the stimulus, i.e., forward relative to the observer), the Y-axis represents the vertical position (superior–inferior), and the Z-axis represents the horizontal position (lateral; left–right). (A) Experiment 2. (B) Experiment 3.
